# ArtemisiaDB: A comprehensive multi-omics database for *Artemisia annua*

**DOI:** 10.1016/j.xplc.2026.101826

**Published:** 2026-03-17

**Authors:** Ayat Taheri, Fabricio Almeida-Silva, Yaojie Zhang, Xueqing Fu, Ling Li, Yuliang Wang, Kexuan Tang

**Affiliations:** 1 Joint International Research Laboratory of Metabolic and Developmental Sciences, Frontiers Science Center for Transformative Molecules, Plant Biotechnology Research Center, Fudan-SJTU-Nottingham Plant Biotechnology R&D Center, School of Agriculture and Biology, Shanghai Jiao Tong University, Shanghai 200240, China; 2 College of Life Sciences and Medicine, Key Laboratory of Plant Secondary Metabolism and Regulation of Zhejiang Province, Zhejiang Sci-Tech University, Hangzhou 310018, China; 3 Department of Plant Biotechnology and Bioinformatics, Ghent University, 9052 Ghent, Belgium; 4 VIB Center for Plant Systems Biology, VIB, 9052 Ghent, Belgium; 5 School of Design, Shanghai Jiao Tong University, Shanghai 200240, China; 6 Shanghai Jiao Tong University Sichuan Research Institute, Chengdu 610213, China; 7 Yazhouwan National Laboratory, No. 8 Huanjin Road, Yazhou District, Sanya City 572024, China

Dear Editor,

*Artemisia annua*, commonly known as sweet wormwood, has profoundly impacted global health through its production of artemisinin, a compound that has revolutionized malaria treatment (Hassani et al., 2023). Beyond its established role in combating malaria, artemisinin and its derivatives show promise in treating various other diseases, including cancer and diabetes, highlighting their broad therapeutic potential (Li et al., 2024). However, the naturally low production of artemisinin in *A. annua* presents a significant barrier to meeting global demand. This challenge has driven extensive efforts to enhance artemisinin production through metabolic engineering and genetic optimization. Achieving these goals requires a deeper understanding of the regulatory mechanisms governing artemisinin biosynthesis; therefore, an integrative, multi-omics approach is essential for elucidating these complex processes.

Here, we present ArtemisiaDB (https://artemisia-db.com), a comprehensive, user-friendly, and interactive multi-omics database developed to accelerate *A. annua* research. ArtemisiaDB integrates curated genomic, transcriptomic, and metabolomic data, enabling researchers to explore gene expression patterns, co-expression networks, metabolite associations, and candidate gene functions within a unified platform.

Unlike existing plant databases such as PlantExp and IMP (Liu et al., 2023; Chen et al., 2024), which either exclude *A. annua* or rely on limited and outdated datasets (for example, only nine RNA sequencing [RNA-seq] samples based on the first draft genome), ArtemisiaDB represents a substantial advancement in both data resolution and functional capacity (Supplemental Table 1). Our platform integrates 317 RNA-seq samples sourced from the NCBI Sequence Read Archive, the National Genomics Data Center, and the Global Pharmacopoeia Genome Database as of October 2025 (Supplemental Table 2). These datasets were aligned to the latest chromosome-level *A. annua* genome (LQ9-Phase0) (Liao et al., 2022) using a Mikado-refined assembly pipeline. In addition, ArtemisiaDB incorporates specialized modules for TF analysis, CRISPR sgRNA design, and gene–metabolite correlation analysis, providing a level of resolution and functionality previously unavailable for this species.

To ensure continued relevance, ArtemisiaDB will be updated annually to incorporate newly published RNA-seq, metabolomic, and functional data through standardized curation and quality-control pipelines (Figure 1A). As shown in Figure 1B, initial quality control revealed Q20 rates predominantly ranging from 97.5% to 99.5%, indicating high sequencing quality and a bimodal distribution of total bases. Low-quality reads (mean length < 40 bp or Q20 < 80%) were removed during preprocessing, while all RNA-seq datasets met the overall quality criteria and were retained for downstream analysis. Following rigorous quality control, these datasets were aligned to the *A. annua* genome, yielding a mean alignment rate exceeding 80% (supplemental methods). To maximize the utility of these extensive data, we constructed a comprehensive transcriptome by integrating annotations from multiple tools. The assembly was generated by combining both short- and long-read RNA-seq data using StringTie (Pertea et al., 2015) and IsoQuant (Prjibelski et al., 2023). The final transcriptome, processed through the Mikado pipeline (Venturini et al., 2018) and de-duplicated with MMseqs2 (Mirdita et al., 2019), demonstrated high completeness (97.6% BUSCO genes, based on the embryophyta_odb10 database) and minimal redundancy, providing an accurate reference for subsequent analyses (Figure 1C). This robust transcriptome assembly facilitates deeper insights into the regulatory and metabolic pathways in *A. annua*, including artemisinin biosynthesis.

**Figure 1:**
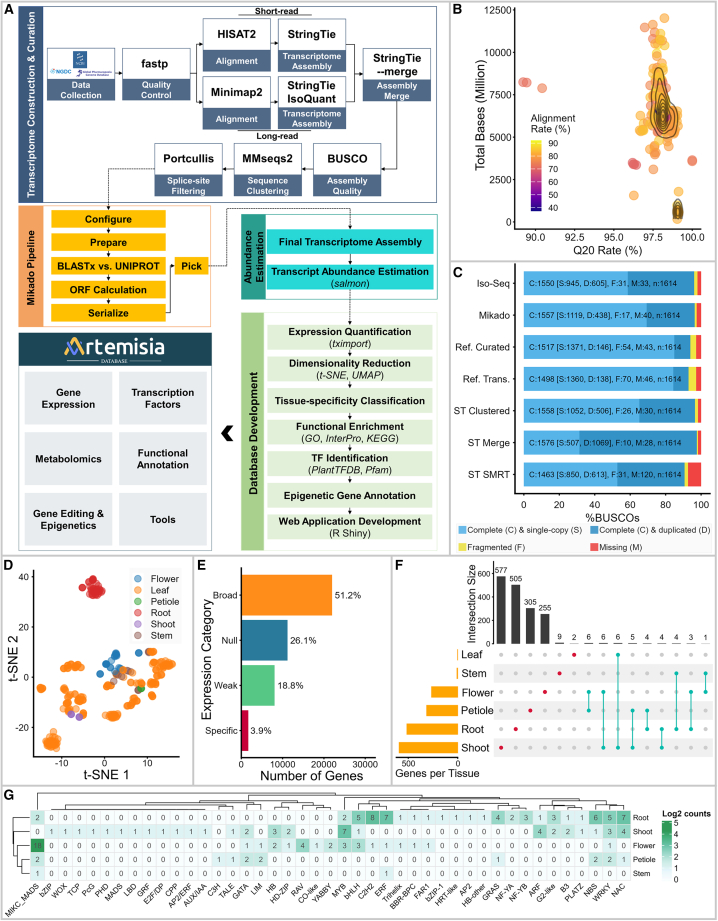
Comprehensive overview of ArtemisiaDB development, quality control, and transcriptome characterization. **(A)** Workflow of ArtemisiaDB development and analysis. **(B)** Scatter plot of Q20 rate (%) vs. total bases (million), with alignment rates (%) indicated by color, and density contours for RNA-seq samples. **(C)** BUSCO summary assessing assembly completeness. ST, StringTie; Ref. Curated, manually curated reference transcriptome; Ref. Trans., reference transcriptome; ST Clustered, StringTie assemblies clustered with MMseqs2; ST SMRT, StringTie assemblies integrating SMRT long-read data. **(D)** t-distributed stochastic neighbor embedding (t-SNE) plot of RNA-seq samples based on the top 14 principal components for dimensionality reduction. **(E)** Distribution of genes across four expression categories defined by the τ index and median TPM values: null expression (median TPM < 1 in all tissues), weak expression (median TPM < 5 in all tissues), broad expression (τ < 0.85 and median TPM > 5), and tissue-specific expression (τ ≥ 0.85 and median TPM > 5). **(F)** UpSet plot showing the overlap of tissue-specific genes across tissues. **(G)** Heatmap showing the distribution of transcription factor (TF) families among tissue-specific genes identified using the τ index. Numbers indicate the counts of TFs in each family exhibiting tissue-specific expression across five tissues (petiole, leaf, stem, flower, and root). TF families were classified based on PlantTFDB and Pfam annotations.

In-depth analyses using t-distributed stochastic neighbor embedding (Figure 1D) and uniform manifold approximation and projection (Supplemental Figure 1) revealed distinct tissue-specific clustering among the RNA-seq samples. Root samples formed a tight cluster, whereas leaf samples exhibited greater dispersion. This variability suggests a more dynamic leaf transcriptome, likely reflecting its responsiveness to environmental cues and developmental stages. We quantified tissue specificity using the τ index, classifying genes into four categories—null, weak, broad, and specific (Almeida-Silva et al., 2023) (Figure 1E). A total of 1692 genes exhibited tissue-specific expression, with shoot samples harboring the largest number (Figure 1F). These results indicate that shoots, together with roots and petioles, possess distinct gene sets, highlighting tissue-specific transcriptional patterns.

We identified TFs among these tissue-specific genes using BLASTx against the PlantTFDB and Pfam databases (Figure 1G). The results show that shoot and root tissues possess a high diversity of TF families, with 25 families identified in shoots and 22 in roots. In contrast, stem and petiole tissues contain fewer tissue-specific TFs. Functional enrichment analysis further revealed distinct roles of these genes in secondary metabolism, defense responses, and TF distribution. For instance, root-specific genes were significantly enriched in pathways implicated in defense and specialized metabolism, including the cytochrome P450 superfamily (39 genes, IPR001128) and peroxidase activity (13 genes, GO:0004601), both of which are associated with oxidative stress-related metabolic processes. These pathways are essential for the biosynthesis of bioactive compounds and the reinforcement of cell walls during stress, suggesting their potential role in root-associated defense processes.

ArtemisiaDB is structured into several intuitive modules: (1) gene expression, (2) TFs, (3) metabolomics, (4) functional annotation, (5) gene editing and epigenetics, (6) tools, and (7) download.

The gene expression module provides insights into the global expression landscape of Artemisia and its associated metadata. Users can examine median gene expression across tissues, visualize the expression of genes involved in the artemisinin biosynthesis pathway, and categorize genes based on the specificity index.

The TF module offers detailed information on TF families identified through PlantTFDB and Pfam. Users can investigate expression patterns and retrieve sequences for TFs of interest. Additionally, an interactive heatmap enables visualization of tissue-specific TF distribution and their expression across different tissues.

The metabolomics module provides direct correlations between gene expression and metabolite abundance in *A. annua*, supporting functional genomics studies. It enables the identification of potential biosynthetic pathways and candidate genes by exploring co-expression and co-occurrence patterns. The module also offers downloadable datasets for further computational analysis and experimental validation.

The functional annotation module allows users to query multiple annotation datasets, including GO terms and Pfam domains. Users can upload gene lists for annotation analysis or retrieve gene information by searching specific functional terms.

The gene editing and epigenetics module is divided into two sections. The CRISPR sgRNA design section allows users to explore predicted target sites for gene editing, while the methylation section provides access to epigenetic data, such as N6-methyladenosine modifications.

Beyond the core modules, the tools module provides powerful functionalities for genomic and functional analyses. JBrowse offers interactive visualization of genomic features, allowing users to explore gene annotations and sequences. BLAST supports sequence similarity searches to identify corresponding Artemisia gene IDs and integrates seamlessly with other database features. The co-expression analysis tool identifies co-expressed genes across BioProjects and plant tissues, with customizable correlation methods and enrichment visualizations. The GO enrichment tool enables identification of enriched biological terms for user-defined gene lists, while the get gene sequences tool enables easy retrieval and download of gene sequences.

The download module ensures seamless access to raw data for advanced genomic analyses. Users can download gene expression matrices organized by tissue in formats such as TPM or bias-corrected counts at either the gene or transcript level. The download by project feature allows retrieval of data from individual BioProjects. In addition, the latest annotation and latest transcriptome assembly options provide direct access to the study’s GFF3 annotation and FASTA transcriptome assembly files, respectively, supporting comprehensive downstream analyses.

A compelling case study focusing on 3-hydroxy-3-methylglutaryl-CoA reductase, a key enzyme in the artemisinin biosynthesis pathway (Li et al., 2024), illustrates the utility of ArtemisiaDB (Supplemental Figures 2 and 3). Co-expression analysis in leaf tissue identified 11 hormone-responsive genes, eight of which were TFs belonging to the auxin response factor (ARF) family. This finding suggests a novel regulatory link between auxin signaling and artemisinin biosynthesis. Although this finding is correlative, increasing experimental evidence supports a role for auxin signaling—and ARFs in particular—in both trichome development and artemisinin biosynthesis in *A. annua*. For example, overexpression of the auxin biosynthesis gene *iaaM* significantly increases the density, length, and width of glandular secretory trichomes, along with elevated artemisinin content, suggesting that auxin promotes both trichome development and the metabolic pathways involved in artemisinin production (Zhao et al., 2024). In addition, the miR160–ARF1 regulatory module has been shown to control glandular secretory trichome formation and activate the expression of *DBR2*, a key gene in the artemisinin biosynthesis pathway, suggesting that ARFs function as upstream regulators of both trichome development and metabolite production (Guo et al., 2023). A plausible mechanism is that ARFs bind to auxin-responsive promoter elements upstream of biosynthetic genes such as *HMGR* or *DBR2*, thereby linking hormonal signaling to transcriptional regulation. Future validation using chromatin immunoprecipitation, promoter–reporter assays, or functional analyses of ARF overexpression and silencing lines will be essential to confirm these interactions. This case study demonstrates how ArtemisiaDB can be used to generate testable hypotheses and identify regulatory components to guide metabolic engineering strategies for enhanced artemisinin production.

Looking ahead, ArtemisiaDB is designed to support future multi-omics expansion. Planned updates include the integration of single-cell and spatial transcriptomics, which will enable cell-type-specific exploration of biosynthetic pathways, as well as epigenomic layers—such as DNA methylation and histone modification data—to better elucidate the regulatory mechanisms underlying gene expression. Moreover, the incorporation of metabolite flux and isotope-labeling data will allow quantitative integration of transcriptomic and metabolomic dynamics. These developments will extend ArtemisiaDB from a descriptive resource toward a more predictive framework for understanding and engineering *A. annua* metabolism.

In conclusion, ArtemisiaDB provides a valuable resource for advancing our understanding of the molecular mechanisms governing artemisinin biosynthesis in *A. annua*. By providing an integrated platform for high-quality multi-omics data, ArtemisiaDB facilitates efforts to optimize artemisinin production and advance plant metabolic engineering. We believe this database will be instrumental for researchers working on both fundamental and applied research on *A. annua*.

## Data and code availability

The datasets underpinning this study are available in the aforementioned online repositories. All code used for the analyses is accessible via the following GitHub repository: https://github.com/ayattaheri/artemisia-db. ArtemisiaDB (https://artemisia-db.com) will be updated annually to incorporate newly published RNA-seq, metabolomic, and functional datasets, with each update version documented on the database website.

## Funding

This work was supported by the Shanghai Municipal Science and Technology Major Project; the National Natural Science Foundation of China (82274047); the Bill & Melinda Gates Foundation (INV-027291); and the National Key Research and Development Program of China (2023YFC3503901).

## Acknowledgments

In accordance with the grant conditions of the Bill & Melinda Gates Foundation, any author-accepted manuscript arising from this submission will be made available under a Creative Commons Attribution 4.0 Generic License. The computations presented in this study were performed on the Siyuan-1 cluster, supported by the Center for High Performance Computing at Shanghai Jiao Tong University. We acknowledge the valuable contributions of the researchers and organizations who generated the genome and RNA-seq data that made this study possible. The authors declare no competing interests.

## Author contributions

A.T. conceived the study, performed data analyses, developed the web application, and drafted the manuscript. F.A.-S. contributed to data analysis, manuscript revision, and discussion. X.F. and K.T. provided funding support. K.T. supervised the study and serves as the corresponding author. Y.Z., L.L., and Y.W. contributed to manuscript revision and discussion. All authors reviewed and approved the final manuscript.

## Supplemental information

Document S2. Supplemental Tables 1–4

Document S3. Article plus supplemental information
