# Supplementary material for: ArtemisiaDB: A comprehensive multi-omics database for Artemisia annua
Source: Plant Commun. 2026 Mar 17;7(7):101826. doi: 10.1016/j.xplc.2026.101826 (PMC13370211; doi:10.1016/j.xplc.2026.101826)
Supplement: Document S1. Supplemental Figures 1–6 and supplemental methods [file mmc1.pdf]

Plant Communications, Volume 7

Supplemental information

ArtemisiaDB: A comprehensive multi-omics database for *Artemisia annua*

Ayat Taheri, Fabricio Almeida-Silva, Yaojie Zhang, Xueqing Fu, Ling Li, Yuliang Wang, and Kexuan Tang

ArtemisiaDB: A Comprehensive Multi-Omics Database for *Artemisia annua*

Ayat Taheri[1,2], Fabricio Almeida-Silva[3,4], Yaojie Zhang[1], Xueqing Fu[5], Ling Li[1], Yuliang Wang[1,6], Kexuan Tang[1,7*]

1. Joint International Research Laboratory of Metabolic and Developmental Sciences, Frontiers Science Center for Transformative Molecules, Plant Biotechnology Research Center, Fudan-SJTU-Nottingham Plant Biotechnology R&D Center, School of Agriculture and Biology, Shanghai Jiao Tong University, Shanghai 200240, China
2. College of Life Sciences and Medicine, Key Laboratory of Plant Secondary Metabolism and Regulation of Zhejiang Province, Zhejiang Sci-Tech University, Hangzhou 310018, China
3. Department of Plant Biotechnology and Bioinformatics, Ghent University, 9052 Ghent, Belgium
4. VIB Center for Plant Systems Biology, VIB, 9052 Ghent, Belgium
5. School of Design, Shanghai Jiao Tong University, Shanghai 200240, China
6. Shanghai Jiao Tong University Sichuan Research Institute, Chengdu, 610213, China
7. Yazhouwan National Laboratory, No. 8 Huanjin Road, Yazhou District, Sanya City 572024, China

***Corresponding author:**

Kexuan Tang, E-mail: kxtang@sjtu.edu.cn

Methods

Data Sources and Preprocessing

For building the ArtemisiaDB, SRA files of RNA-Seq data available on the NCBI's Sequence Read Archive (SRA), National Genomics Data Center (https://ngdc.cncb.ac.cn/) and Global Pharmacopeia Genome Database (http://www.gpgenome.com/) databases up to 2025-10-01 (317 samples) along with the latest available genome of *A. annua* (LQ9-Phase0) (Liao et al., 2022) were downloaded (Supplemental table 1). Gene expression profiling was conducted using a combination of paired-end and single-end short-read RNA-seq data. To improve the accuracy of our transcriptome assembly, we integrated PacBio Iso-Seq full-length transcript reads. Although long-read sequencing is typically employed for genome assembly, Iso-Seq data were specifically used here to capture complete transcript models and improve isoform resolution.

Conversion of SRA files to FASTQ was performed using fastq-dump (3.1.0), which is part of the SRA Toolkit. To ensure the quality of the FASTQ files, fastp (0.23.2) (Chen et al., 2018) was used, and FASTQ files with a mean read length lower than 40 and/or Q20 rate lower than 80%, and the adapter sequences were removed.

Transcriptome Assembly

To construct a comprehensive transcriptome for *A. annua*, we integrated both short-read and long-read RNA-Seq data. Short-read sequences were aligned using HISAT2 (v2.2.1), while long-read sequences were aligned with Minimap2 (v2.17). Samples with an overall alignment rate below 50% were excluded from further analysis.

For transcript assembly, StringTie (v2.2.1) (Pertea et al., 2015) was used for short-read data, while both StringTie and IsoQuant were employed for long-read data. The assembled transcripts from short-read sequences were merged using the `stringtie --merge` command to generate a unified transcript set.

Initial quality assessment of the assembled transcriptomes

The quality of the resulting transcriptomes was evaluated to identify the most comprehensive and accurate assembly for downstream analysis. Four assemblies were compared: the reference transcriptome, the StringTie-assembled transcriptome derived from both short-read and long-read RNA-Seq data, and the IsoQuant-assembled transcriptome. To assess assembly completeness and quality, BUSCO (embryophyta_odb10 database, v5.7.1) (Seppey et al., 2019) was used, which evaluates transcriptome assemblies based on the presence of conserved single-

copy orthologs. This initial evaluation provided a foundational understanding of the quality of each assembly, which was essential for developing a scoring system in the Mikado pipeline. By identifying a robust and representative transcriptome, this approach ensured not only a comprehensive assembly but also a framework for prioritizing transcripts and minimizing redundancy in subsequent analyses.

Clustering Using MMseqs2

While the StringTie-assembled transcriptome exhibited the highest number of complete BUSCOs and the lowest number of missing BUSCOs, it also displayed a high number of duplications. This indicated a significant level of redundancy within the assembly. To address this issue, MMseqs2 (Many-against-Many sequence searching tool) was employed to cluster highly similar sequences and reduce redundancy (Steinegger and Söding, 2017). By grouping similar transcripts (--min-seq-id 0.8), the clustering process aimed to refine the assembly, ensuring a more accurate representation of unique genes while maintaining the integrity of biologically relevant sequences.

Splice-site filtering

To improve the accuracy of splice-site detection in the long-read assemblies, Portcullis was employed. Portcullis is a specialized tool designed to identify and filter splice junctions with high precision, addressing errors commonly found in transcriptome assemblies generated from long-read sequencing data (Mapleson et al., 2018). By applying this filtering step, erroneous or low-confidence splice sites were removed, resulting in a cleaner and more reliable set of splice junctions. This refinement was crucial for ensuring the quality of the transcriptome assembly and for minimizing false positives that could impact downstream analyses, including transcript annotation and functional characterization.

Merging annotations using the Mikado pipeline

To integrate and refine the four transcriptome assemblies—reference transcriptome, StringTie (short-read and long-read), and IsoQuant—we used the Mikado pipeline (Venturini et al., 2018). Mikado is designed to identify the most accurate and representative set of transcripts from multiple assembly methods. The pipeline first defines gene loci based on overlap criteria and evaluates each transcript within a locus using up to 50 metrics, including ORF and cDNA size, UTR length, and the relative position of the ORF. Mikado also utilizes BLASTX-based protein similarity and can integrate junction confidence data from Portcullis to improve splice-site accuracy. The best-scoring transcripts are selected as primary gene models, while valid splice

variants are retained when compatible with the primary isoform. This approach ensures that the final set of transcripts minimizes redundancy, improves gene model quality, and enhances the representation of expressed loci. Mikado's ability to integrate data from both short-read and long-read technologies allowed us to merge assemblies generated through different approaches, resulting in a more comprehensive and accurate transcriptome for further downstream analysis.

Mikado Assembly Validation

To evaluate the Mikado-based assembly, we benchmarked its performance against the reference and manually-curated transcriptomes. Biological completeness was assessed using BUSCO v5.7.1 against the embryophyta_odb10 lineage, while structural continuity and ORF content were analyzed using Transrate (Smith-Unna et al., 2016). Functional depth was quantified by identifying transcripts with ≥ 90% protein coverage via Trinity (Grabherr et al., 2011), and assembly accuracy was validated through rnaQUAST (Bushmanova et al., 2016)metrics and Reads Mapped Back (RMBT) percentages (Langmead and Salzberg, 2012). Overall, the Mikado assembly demonstrated superior quality across nearly all evaluated metrics, justifying its selection as the core reference for ArtemisiaDB (Supplemental Table 4).

Gene-level transcript abundance

After generating the representative assembly for *A. annua*, transcript abundances were estimated using the 'mapping-based' mode of Salmon (v1.10.0) with the '--dumpEq' option, which produces the equivalence classes that can be used in differential transcript usage analysis (Patro et al., 2017). Variations in library size across samples are taken into consideration by transcripts per million (TPM). However, TPM does not take variations in transcript length into consideration, meaning that, even when transcripts are expressed at the same level, longer transcripts will typically have higher TPM values than shorter transcripts. To obtain the gene-level transcript abundances, the bias-corrected counts without an offset method from tximport R package was used (Soneson et al., 2016). The "lengthScaledTPM" argument in tximport adjusts for differences in both library size and transcript length across samples. For the transcript-level transcript abundance estimates, the "dtuScaledTPM" argument was used, which first scales using the median transcript length among isoforms of a gene, and then by library size.

Dimensionality reduction

Because bioinformatics data contains numerous attributes (variables), they are naturally high-dimensional. This high dimensionality can present major hurdles for data analysis, obscuring valuable biological insights and complicating downstream analysis. Therefore, appropriate procedures should be taken to boost the biological significance of the data by reducing the number of attributes using dimensionality reduction techniques (Cao et al., 2020). For transcriptomics in particular, dimensionality reduction helps to mitigate noise and enhance the signal from the biological data. To achieve this goal, techniques such as principal component analysis (PCA), t-distributed stochastic neighbor embedding (t-SNE) (Van der Maaten and Hinton, 2008), and uniform manifold approximation and projection (UMAP) (McInnes et al., 2020) are commonly employed. In the present study, we used scran R package (Lun et al., 2016). After log-transforming the gene counts normalized by library size, we extracted the top 3000 genes with the highest biological components to model the mean-variance relationships. Following performing PCA analysis, 14 principal components, which accounted for 70% of the variation, were selected for dimensionality reduction using the t-SNE and UMAP algorithms. Six different perplexity values (5, 10, 15, 20, 25, 30) for t-SNE and six different numbers of nearest neighbors (10, 20, 30, 40, 50, 60) for UMAP were tested. The optimal values were determined based on visual inspection (Supplemental Figure 4A-C).

Identification of broadly expressed and tissue-specific genes

Identifying broadly expressed and tissue-specific genes is crucial for understanding gene function and regulation. Broadly expressed genes often play essential roles in basic cellular processes, while tissue-specific genes are typically involved in specialized tissue functions. Analyzing these expression patterns helps researchers understand gene functions and regulatory mechanisms (Das and Bansal, 2019). To achieve this, we calculated the tissue specificity index (τ index) for each gene by using a log-transformed matrix of the median TPM values per tissue (Yanai et al., 2005). Based on the criteria established by Lüleci and Yılmaz (2022), genes were categorized into four groups according to their τ index and median TPM values: (1) Null expression: genes with median TPM < 1 in all tissues, indicating no expression; (2) Weak expression: genes with median TPM < 5 in all tissues; (3) Broad expression: genes with $\tau < 0.85$ and median TPM > 5; and (4) Tissue-specific expression: genes with $\tau \geq 0.85$ and median TPM > 5 (Supplemental Figure 4D).

Functional annotation of the *A. annua* transcriptome

To create a unified database for the functional annotation of the *A. annua* transcriptome, several analyses were performed. Transcription factors (TFs) were identified using two approaches: first, by performing a BLASTx search against a collection of TFs from the PlantTFDB (Jin et al., 2017), and second, by extracting PFAM IDs related to 63 different TF families through InterProScan (Supplemental Figure 5). For the BLASTx analysis, Diamond was used with the following parameters: --ultra-sensitive --id 60 --evalue 1e-5 --min-score 150 (Buchfink et al., 2021). EggNOG-mapper (2.1.12) (Cantalapiedra et al., 2021) using the HMMER3 mode was used to annotate orthologous groups and functional categories. InterPro domains, Gene Ontology (GO) terms, and KEGG pathways were assigned using InterProScan (5.67-99.0) (Supplemental Figure 6); The KEGG ORTHOLOGY (KO) database (https://www.genome.jp/kegg/ko.html) was used to obtain KO descriptions. Additionally, BLASTx against *Arabidopsis thaliana* protein-coding genes (Araport11 release, 2022-09-15) and the Uniprot database (release 01.03.2023) was performed to further enhance the functional characterization of the transcriptome. To facilitate genome editing, we integrated a design tool powered by the CrisprDesign R package (1.12.0), which generates high-fidelity CRISPR sgRNA candidates with optimized targeting parameters (Hoberecht et al., 2022). A list of artemisinin-related genes was obtained from recently published research (Li et al., 2024). Their sequences were retrieved from the NCBI database, and the corresponding gene IDs in the current annotation were identified using BLASTn for further analysis. For enrichment analysis, enricher() function of the clusterProfiler package was utilized, with all expressed genes serving as the background set.

Development of the web application

The web application was developed using the Shiny framework (Chang et al., 2021), with a Bootstrap-based layout. A MySQL database was used to manage data associated with various sections of the application, ensuring well-organized data retrieval and storage. A partitioned Parquet directory was employed to store the expression database, with the arrow R package (Richardson et al., 2022) providing an efficient interface between R and the Apache Arrow platform. For data visualization, the application leveraged the ggplot2 (Wickham, 2016), plotly (Inc, 2015), and ComplexHeatmap (Gu, 2022) packages.

References

Buchfink, B., Reuter, K., and Drost, H.-G. (2021). Sensitive protein alignments at tree-of-life scale using DIAMOND. *Nat. Methods* **18**:366–368. https://doi.org/10.1038/s41592-021-01101-x.

Bushmanova, E., Antipov, D., Lapidus, A., Suvorov, V., and Prjibelski, A. D. (2016). rnaQUAST: a quality assessment tool for de novo transcriptome assemblies. *Bioinforma. Oxf. Engl.* **32**:2210–2212. https://doi.org/10.1093/bioinformatics/btw218.

Cantalapiedra, C. P., Hernández-Plaza, A., Letunic, I., Bork, P., and Huerta-Cepas, J. (2021). eggNOG-mapper v2: Functional Annotation, Orthology Assignments, and Domain Prediction at the Metagenomic Scale. *Mol. Biol. Evol.* **38**:5825–5829. https://doi.org/10.1093/molbev/msab293.

Cao, Y., Geddes, T. A., Yang, J. Y. H., and Yang, P. (2020). Ensemble deep learning in bioinformatics. *Nat. Mach. Intell.* **2**:500–508. https://doi.org/10.1038/s42256-020-0217-y.

Chang, W., Cheng, J., Allaire, J., Sievert, C., Schloerke, B., Xie, Y., Allen, J., McPherson, J., Dipert, A., and Borges, B. (2021). shiny: Web Application Framework for R. 2021. R package version 1.6. 0. *Ref. Source* Advance Access published 2021.

Chen, S., Zhou, Y., Chen, Y., and Gu, J. (2018). fastp: an ultra-fast all-in-one FASTQ preprocessor. *Bioinformatics* **34**:i884–i890. https://doi.org/10.1093/bioinformatics/bty560.

Das, S., and Bansal, M. (2019). Variation of gene expression in plants is influenced by gene architecture and structural properties of promoters. *PLoS ONE* **14**:e0212678. https://doi.org/10.1371/journal.pone.0212678.

Grabherr, M. G., Haas, B. J., Yassour, M., Levin, J. Z., Thompson, D. A., Amit, I., Adiconis, X., Fan, L., Raychowdhury, R., Zeng, Q., et al. (2011). Trinity: reconstructing a full-length transcriptome without a genome from RNA-Seq data. *Nat. Biotechnol.* **29**:644–652. https://doi.org/10.1038/nbt.1883.

Gu, Z. (2022). Complex heatmap visualization. *iMeta* **1**:e43. https://doi.org/10.1002/imt2.43.

Hoberecht, L., Perampalam, P., Lun, A., and Fortin, J.-P. (2022). A comprehensive Bioconductor ecosystem for the design of CRISPR guide RNAs across nucleases and technologies. *Nat. Commun.* **13**:6568. https://doi.org/10.1038/s41467-022-34320-7.

Inc, P. (2015). Collaborative data science, Montreal, QC: Plotly Technologies Inc Advance Access published 2015.

Jin, J., Tian, F., Yang, D.-C., Meng, Y.-Q., Kong, L., Luo, J., and Gao, G. (2017). PlantTFDB 4.0: toward a central hub for transcription factors and regulatory interactions in plants. *Nucleic Acids Res.* **45**:D1040–D1045. https://doi.org/10.1093/nar/gkw982.

Langmead, B., and Salzberg, S. L. (2012). Fast gapped-read alignment with Bowtie 2. *Nat. Methods* **9**:357–359. https://doi.org/10.1038/nmeth.1923.

Li, Y., Yang, Y., Li, L., Tang, K., Hao, X., and Kai, G. (2024). Advanced metabolic engineering strategies for increasing artemisinin yield in *Artemisia annua* L. *Hortic. Res.* **11**:uhad292. https://doi.org/10.1093/hr/uhad292.

Liao, B., Shen, X., Xiang, L., Guo, S., Chen, S., Meng, Y., Liang, Y., Ding, D., Bai, J., Zhang, D., et al. (2022). Allele-aware chromosome-level genome assembly of *Artemisia annua* reveals the correlation between ADS expansion and artemisinin yield. *Mol. Plant* **15**:1310–1328. https://doi.org/10.1016/j.molp.2022.05.013.

Lüleci, H. B., and Yılmaz, A. (2022). Robust and rigorous identification of tissue-specific genes by statistically extending tau score. *BioData Min.* **15**:31. https://doi.org/10.1186/s13040-022-00315-9.

Lun, A. T. L., McCarthy, D. J., and Marioni, J. C. (2016). A step-by-step workflow for low-level analysis of single-cell RNA-seq data with Bioconductor. *F1000Research* **5**:2122. https://doi.org/10.12688/f1000research.9501.2.

Mapleson, D., Venturini, L., Kaithakottil, G., and Swarbreck, D. (2018). Efficient and accurate detection of splice junctions from RNA-seq with Portcullis. *GigaScience* **7**:giy131. https://doi.org/10.1093/gigascience/giy131.

McInnes, L., Healy, J., and Melville, J. (2020). UMAP: Uniform Manifold Approximation and Projection for Dimension Reduction Advance Access published September 17, 2020.

Patro, R., Duggal, G., Love, M. I., Irizarry, R. A., and Kingsford, C. (2017). Salmon provides fast and bias-aware quantification of transcript expression. *Nat. Methods* **14**:417–419. https://doi.org/10.1038/nmeth.4197.

Pertea, M., Pertea, G. M., Antonescu, C. M., Chang, T.-C., Mendell, J. T., and Salzberg, S. L. (2015). StringTie enables improved reconstruction of a transcriptome from RNA-seq reads. *Nat. Biotechnol.* **33**:290–295. https://doi.org/10.1038/nbt.3122.

Richardson, N., Cook, I., Crane, N., Keane, J., François, R., and Ooms, J. (2022). Arrow: integration to apache arrow Advance Access published 2022.

Seppey, M., Manni, M., and Zdobnov, E. M. (2019). BUSCO: assessing genome assembly and annotation completeness. In *Gene prediction*, pp. 227–245. Springer.

Smith-Unna, R., Boursnell, C., Patro, R., Hibberd, J. M., and Kelly, S. (2016). TransRate: reference-free quality assessment of de novo transcriptome assemblies. *Genome Res.* **26**:1134–1144. https://doi.org/10.1101/gr.196469.115.

Soneson, C., Love, M. I., and Robinson, M. D. (2016). Differential analyses for RNA-seq: transcript-level estimates improve gene-level inferences. *F1000Research* **4**:1521. https://doi.org/10.12688/f1000research.7563.2.

Steinegger, M., and Söding, J. (2017). MMseqs2 enables sensitive protein sequence searching for the analysis of massive data sets. *Nat. Biotechnol.* **35**:1026–1028. https://doi.org/10.1038/nbt.3988.

Van der Maaten, L., and Hinton, G. (2008). Visualizing data using t-SNE. *J. Mach. Learn. Res.* **9**.

Venturini, L., Caim, S., Kaithakottil, G. G., Mapleson, D. L., and Swarbreck, D. (2018). Leveraging multiple transcriptome assembly methods for improved gene structure annotation. *GigaScience* **7**. https://doi.org/10.1093/gigascience/giy093.

Wickham, H. (2016). Getting Started with ggplot2. In *ggplot2*, pp. 11–31. Cham: Springer International Publishing.

Supplemental Figures

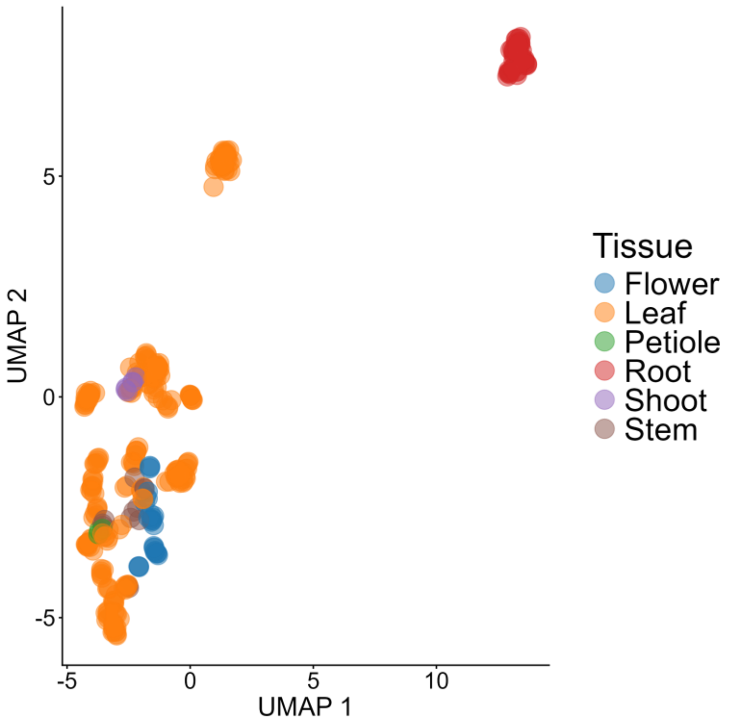

Supplemental Figure 1. UMAP plot of RNA-seq samples based on the top 14 principal components for dimensionality reduction.

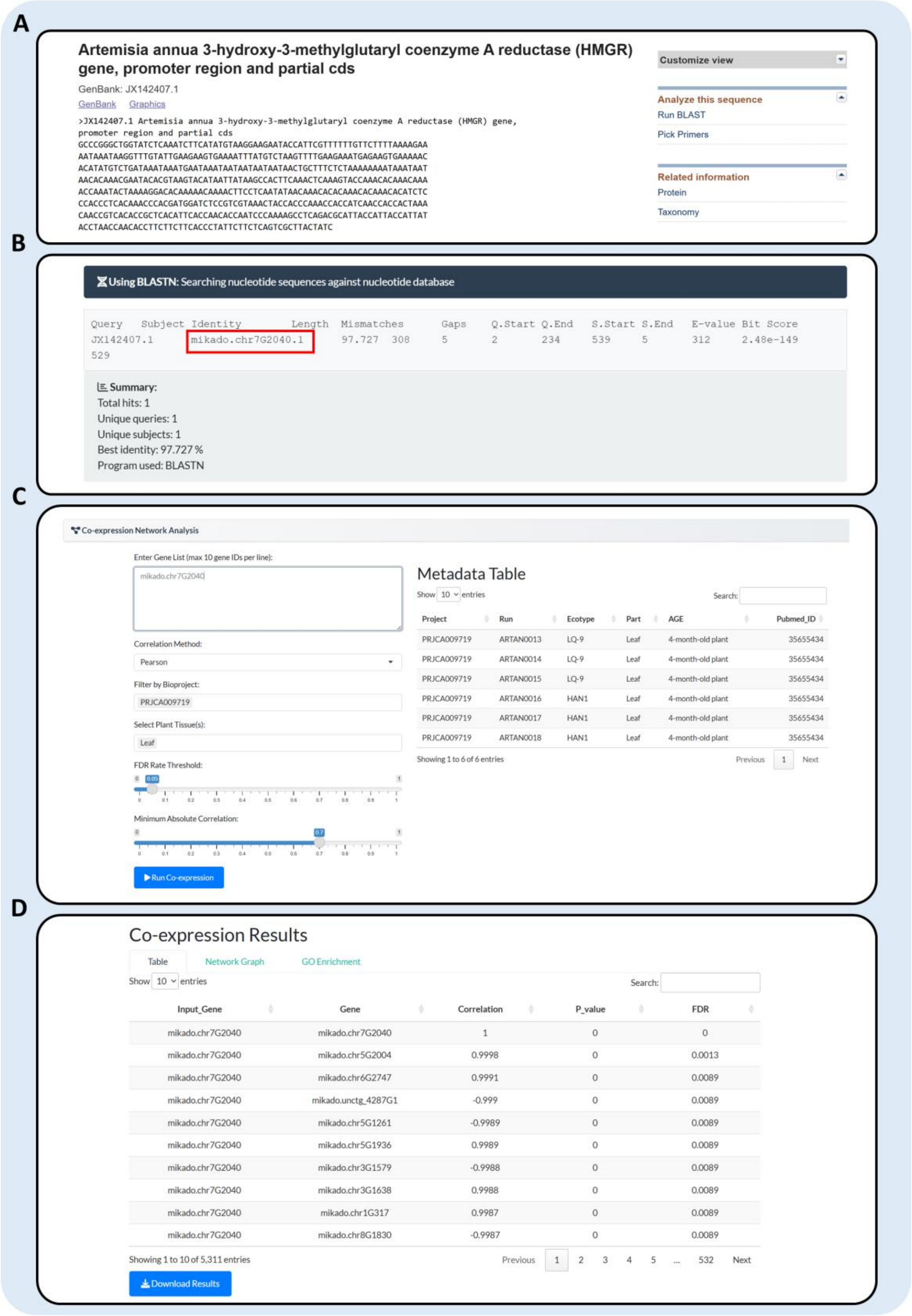

Supplemental Figure 2. Case study of the *HMGR* gene in ArtemisiaDB (Part 1). (A) HMGR gene sequence from NCBI. (B) BLAST results against ArtemisiaDB to identify the corresponding gene ID. (C) Selection of co-expression analysis parameters (Pearson method, leaf samples, FDR ≤ 0.05, |correlation| ≥ 0.7). (D) Co-expression results table.

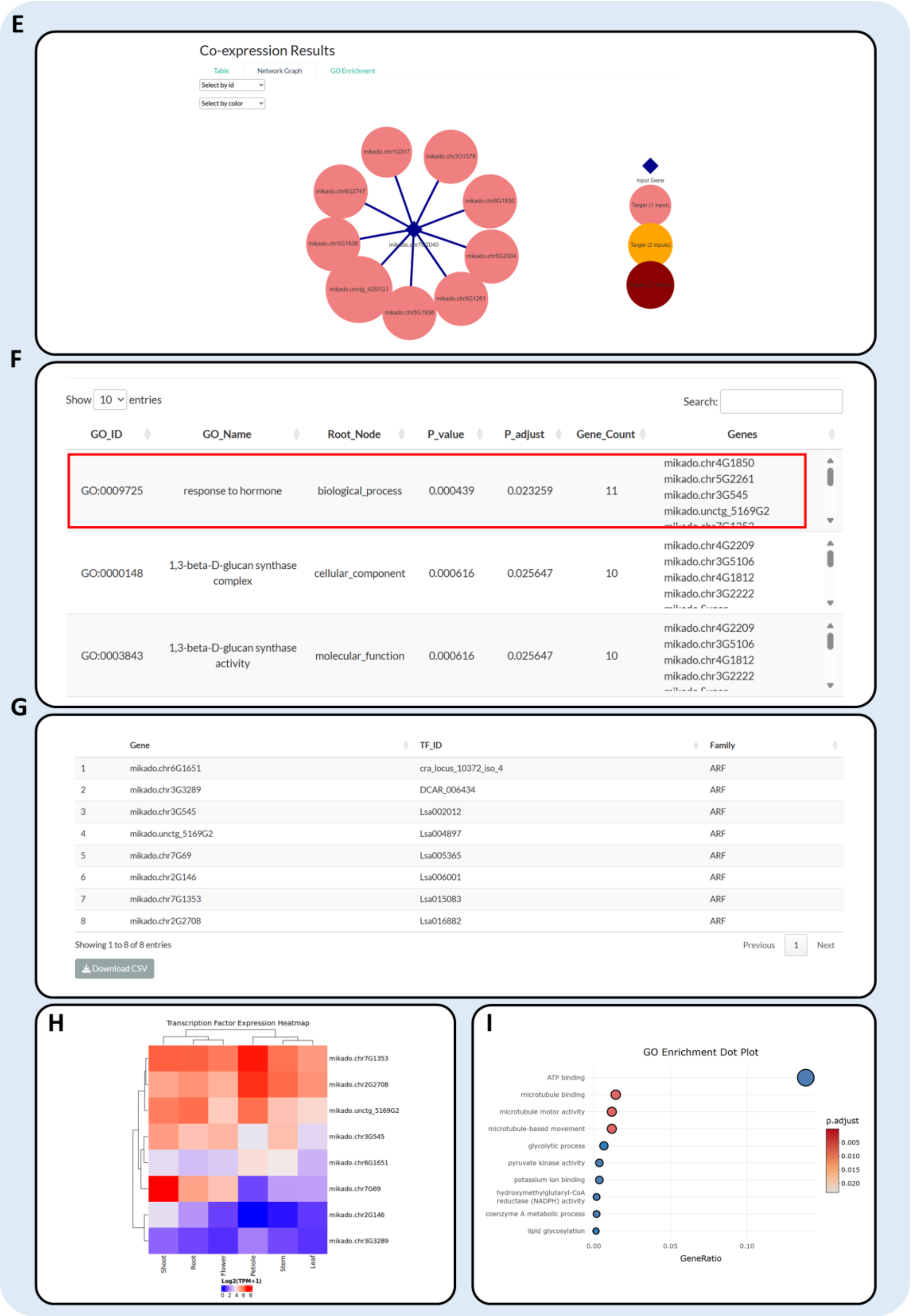

Supplemental Figure 3. Case study of the *HMGR* gene in ArtemisiaDB (Part 2). (E) Co-expression network of the input gene (*HMGR*) and its top 10 co-expressed genes. (F) GO enrichment analysis of the co-expressed genes, highlighting the "response to hormone" term. (G) TFDB search of the co-expressed genes, revealing several ARF transcription factors. (H) Heatmap showing the expression of the identified ARF transcription factors across different tissues. (I) GO enrichment dot plot of the co-expressed genes, showing the most significantly enriched biological process terms.

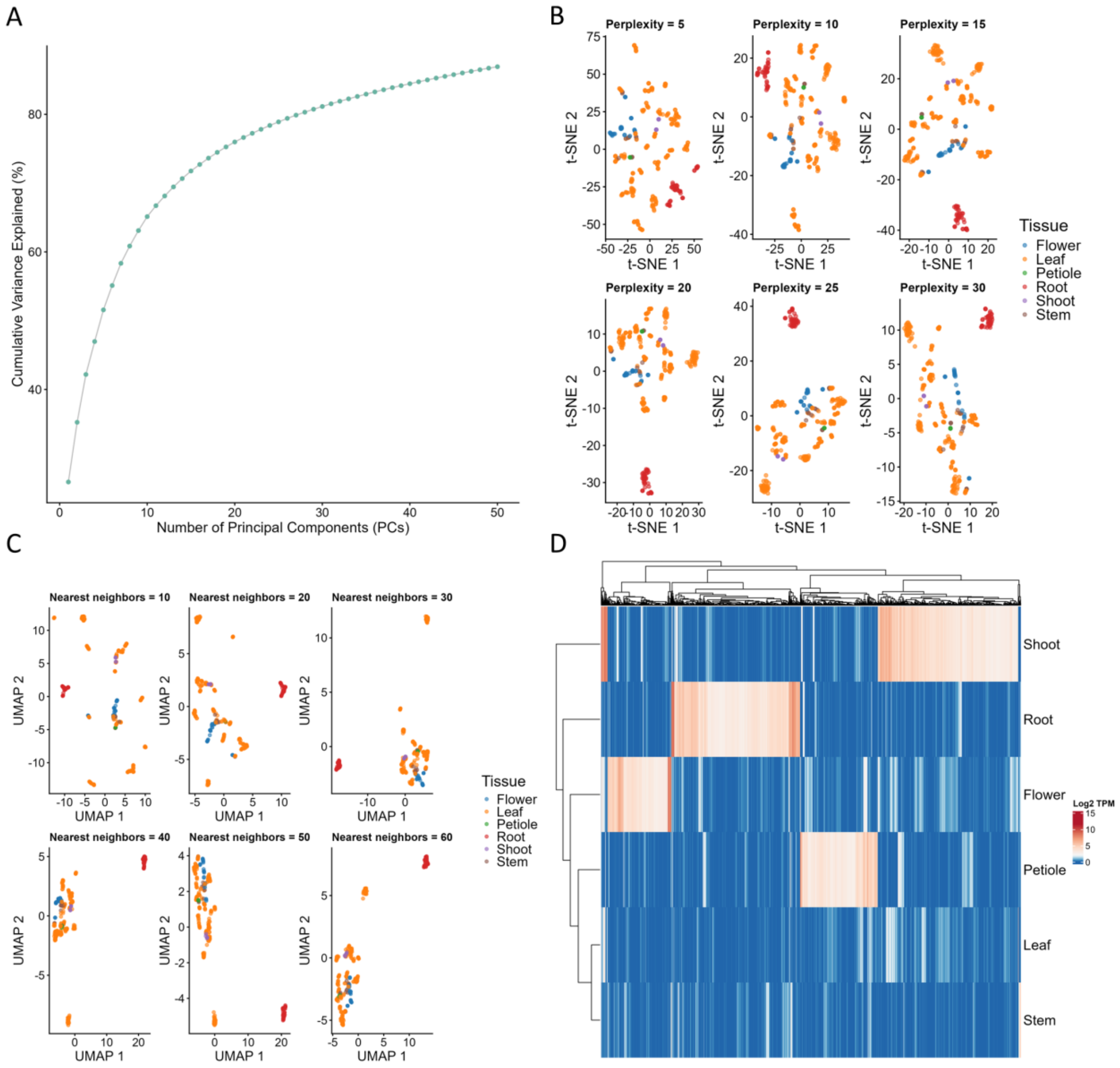

Supplemental Figure 4. (A) Cumulative variance explained by each principal component (PC) in the transcriptome analysis. (B) t-SNE plots generated using different perplexity values (5, 10, 15, 20, 25, 30). (C) UMAP plots based on varying numbers of nearest neighbors (10, 20, 30, 40, 50, 60). (D) Heatmap of median expression levels of tissue-specific genes across different tissues.

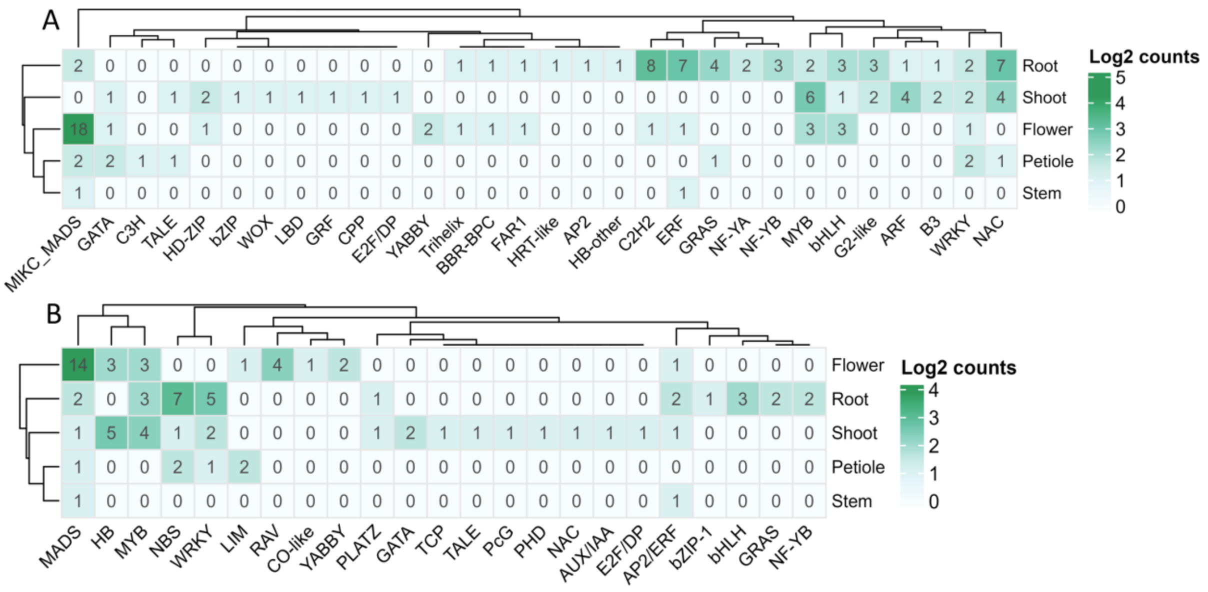

Supplemental Figure 5. Heatmap of transcription factor distribution across tissue-specific genes identified from PlantTFDB. (B) Heatmap of transcription factor distribution across tissue-specific genes identified using Pfam IDs.

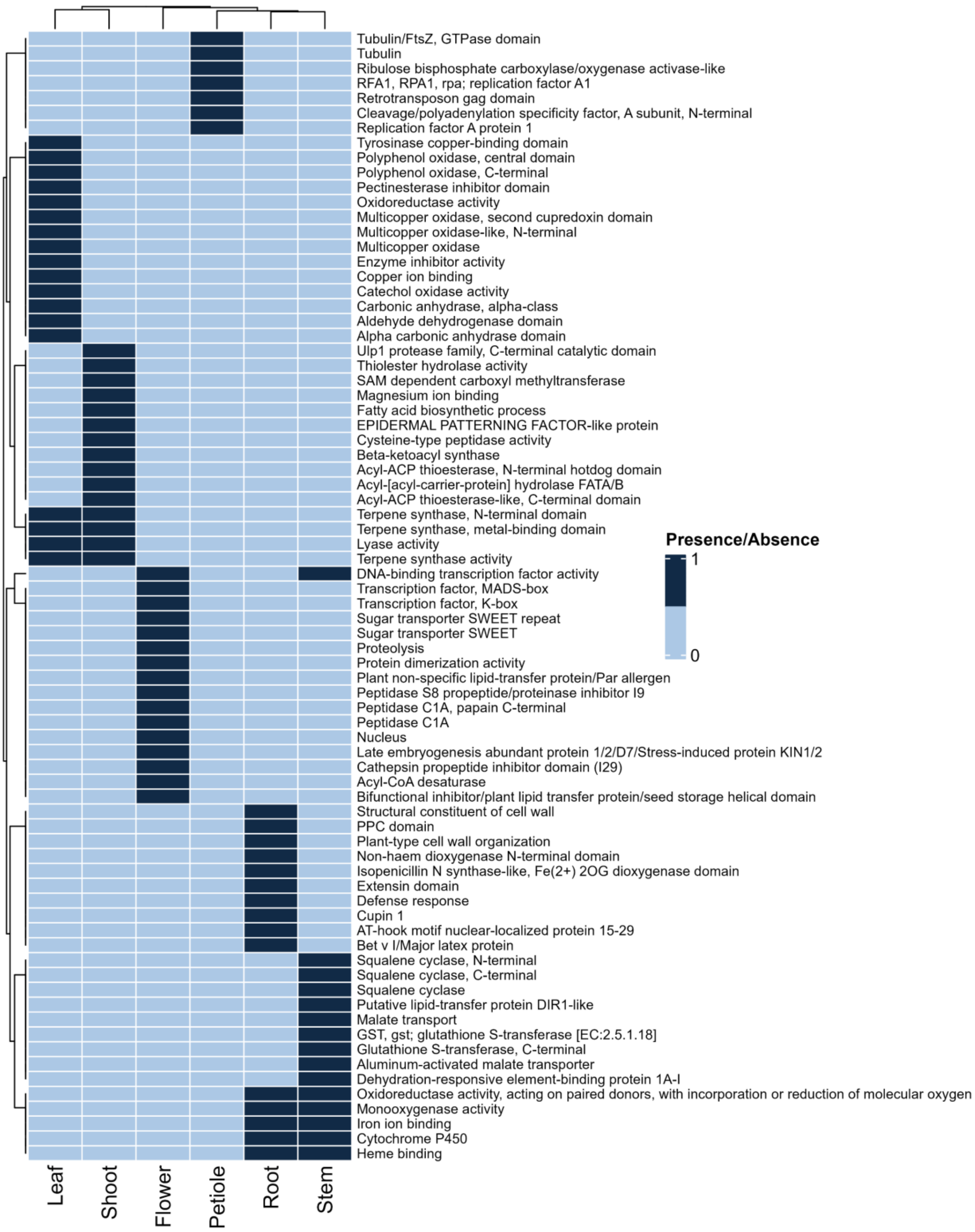

Supplemental Figure 6. Heatmap of Gene Ontology (GO) terms and InterPro domain enrichment for tissue-specific genes.
