# Supplementary material for: ArtemisiaDB: A comprehensive multi-omics database for Artemisia annua
Source: Plant Commun. 2026 Mar 17;7(7):101826. doi: 10.1016/j.xplc.2026.101826 (PMC13370211; doi:10.1016/j.xplc.2026.101826)
Supplement: Document S3. Article plus supplemental information [file mmc3.pdf]

Plant Communications
Correspondence

CellPress
Partner Journal

ArtemisiaDB: A comprehensive multi-omics database for *Artemisia annua*

Dear Editor,

Artemisia annua, commonly known as sweet wormwood, has profoundly impacted global health through its production of artemisinin, a compound that has revolutionized malaria treatment (Hassani et al., 2023). Beyond its established role in combating malaria, artemisinin and its derivatives show promise in treating various other diseases, including cancer and diabetes, highlighting their broad therapeutic potential (Li et al., 2024). However, the naturally low production of artemisinin in *A. annua* presents a significant barrier to meeting global demand. This challenge has driven extensive efforts to enhance artemisinin production through metabolic engineering and genetic optimization. Achieving these goals requires a deeper understanding of the regulatory mechanisms governing artemisinin biosynthesis; therefore, an integrative, multi-omics approach is essential for elucidating these complex processes.

Here, we present ArtemisiaDB (https://artemisia-db.com), a comprehensive, user-friendly, and interactive multi-omics database developed to accelerate *A. annua* research. ArtemisiaDB integrates curated genomic, transcriptomic, and metabolomic data, enabling researchers to explore gene expression patterns, co-expression networks, metabolite associations, and candidate gene functions within a unified platform.

Unlike existing plant databases such as PlantExp and IMP (Liu et al., 2023; Chen et al., 2024), which either exclude *A. annua* or rely on limited and outdated datasets (for example, only nine RNA sequencing [RNA-seq] samples based on the first draft genome), ArtemisiaDB represents a substantial advancement in both data resolution and functional capacity (Supplemental Table 1). Our platform integrates 317 RNA-seq samples sourced from the NCBI Sequence Read Archive, the National Genomics Data Center, and the Global Pharmacopoeia Genome Database as of October 2025 (Supplemental Table 2). These datasets were aligned to the latest chromosome-level *A. annua* genome (LQ9-Phase0) (Liao et al., 2022) using a Mikado-refined assembly pipeline. In addition, ArtemisiaDB incorporates specialized modules for TF analysis, CRISPR sgRNA design, and gene–metabolite correlation analysis, providing a level of resolution and functionality previously unavailable for this species.

To ensure continued relevance, ArtemisiaDB will be updated annually to incorporate newly published RNA-seq, metabolomic, and functional data through standardized curation and quality-control pipelines (Figure 1A). As shown in Figure 1B, initial quality control revealed Q20 rates predominantly ranging from 97.5% to 99.5%, indicating high sequencing quality and a bimodal distribution of total bases. Low-quality reads (mean length < 40 bp or Q20 < 80%) were removed during preprocessing, while all RNA-seq datasets met the overall quality criteria and were retained for downstream analysis. Following rigorous quality control, these datasets were aligned to the *A. annua* genome, yielding a mean alignment rate exceeding 80% (supplemental methods). To maximize the utility of these extensive data, we constructed a comprehensive transcriptome by integrating annotations from multiple tools. The assembly was generated by combining both short- and long-read RNA-seq data using StringTie (Pertea et al., 2015) and IsoQuant (Prjibelski et al., 2023). The final transcriptome, processed through the Mikado pipeline (Venturini et al., 2018) and de-duplicated with MMseqs2 (Mirdita et al., 2019), demonstrated high completeness (97.6% BUSCO genes, based on the embryophyta_odb10 database) and minimal redundancy, providing an accurate reference for subsequent analyses (Figure 1C). This robust transcriptome assembly facilitates deeper insights into the regulatory and metabolic pathways in *A. annua*, including artemisinin biosynthesis.

In-depth analyses using t-distributed stochastic neighbor embedding (Figure 1D) and uniform manifold approximation and projection (Supplemental Figure 1) revealed distinct tissue-specific clustering among the RNA-seq samples. Root samples formed a tight cluster, whereas leaf samples exhibited greater dispersion. This variability suggests a more dynamic leaf transcriptome, likely reflecting its responsiveness to environmental cues and developmental stages. We quantified tissue specificity using the τ index, classifying genes into four categories—null, weak, broad, and specific (Almeida-Silva et al., 2023) (Figure 1E). A total of 1692 genes exhibited tissue-specific expression, with shoot samples harboring the largest number (Figure 1F). These results indicate that shoots, together with roots and petioles, possess distinct gene sets, highlighting tissue-specific transcriptional patterns.

We identified TFs among these tissue-specific genes using BLASTx against the PlantTFDB and Pfam databases (Figure 1G). The results show that shoot and root tissues possess a high diversity of TF families, with 25 families identified in shoots and 22 in roots. In contrast, stem and petiole tissues contain fewer tissue-specific TFs. Functional enrichment analysis further revealed distinct roles of these genes in secondary metabolism, defense responses, and TF distribution. For instance, root-specific genes were significantly enriched in pathways implicated in defense and specialized metabolism, including the cytochrome P450 superfamily (39 genes, IPR001128) and peroxidase activity (13 genes, GO:0004601), both of which are associated with oxidative stress-related metabolic processes. These pathways are essential for the biosynthesis of bioactive compounds and the reinforcement of cell walls during stress, suggesting their potential role in root-associated defense processes.

ArtemisiaDB is structured into several intuitive modules: (1) gene expression, (2) TFs, (3) metabolomics, (4) functional annotation, (5) gene editing and epigenetics, (6) tools, and (7) download.

 © 2026 The Authors. Published by Elsevier Inc. on behalf of CAS Center for Excellence in Molecular Plant Sciences, Chinese Academy of Sciences, and Chinese Society for Plant Biology.
This is an open access article under the CC BY license (http://creativecommons.org/licenses/by/4.0/).

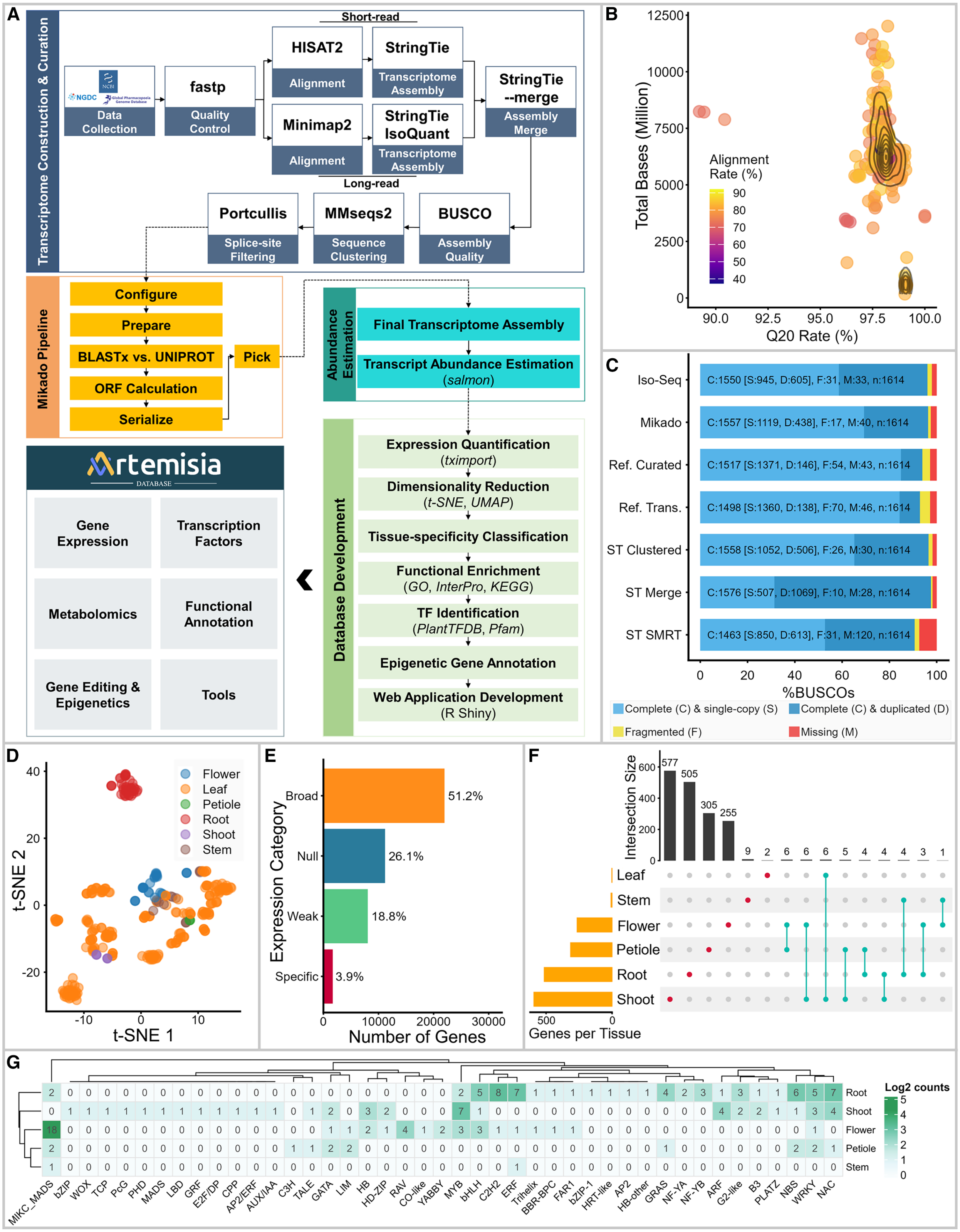

Figure 1. Comprehensive overview of ArtemisiaDB development, quality control, and transcriptome characterization.
(A) Workflow of ArtemisiaDB development and analysis.
(B) Scatter plot of Q20 rate (%) vs. total bases (million), with alignment rates (%) indicated by color, and density contours for RNA-seq samples.

(legend continued on next page)

The gene expression module provides insights into the global expression landscape of Artemisia and its associated metadata. Users can examine median gene expression across tissues, visualize the expression of genes involved in the artemisinin biosynthesis pathway, and categorize genes based on the specificity index.

The TF module offers detailed information on TF families identified through PlantTFDB and Pfam. Users can investigate expression patterns and retrieve sequences for TFs of interest. Additionally, an interactive heatmap enables visualization of tissue-specific TF distribution and their expression across different tissues.

The metabolomics module provides direct correlations between gene expression and metabolite abundance in *A. annua*, supporting functional genomics studies. It enables the identification of potential biosynthetic pathways and candidate genes by exploring co-expression and co-occurrence patterns. The module also offers downloadable datasets for further computational analysis and experimental validation.

The functional annotation module allows users to query multiple annotation datasets, including GO terms and Pfam domains. Users can upload gene lists for annotation analysis or retrieve gene information by searching specific functional terms.

The gene editing and epigenetics module is divided into two sections. The CRISPR sgRNA design section allows users to explore predicted target sites for gene editing, while the methylation section provides access to epigenetic data, such as N6-methyladenosine modifications.

Beyond the core modules, the tools module provides powerful functionalities for genomic and functional analyses. JBrowse offers interactive visualization of genomic features, allowing users to explore gene annotations and sequences. BLAST supports sequence similarity searches to identify corresponding Artemisia gene IDs and integrates seamlessly with other database features. The co-expression analysis tool identifies co-expressed genes across BioProjects and plant tissues, with customizable correlation methods and enrichment visualizations. The GO enrichment tool enables identification of enriched biological terms for user-defined gene lists, while the get gene sequences tool enables easy retrieval and download of gene sequences.

The download module ensures seamless access to raw data for advanced genomic analyses. Users can download gene expression matrices organized by tissue in formats such as TPM or bias-corrected counts at either the gene or transcript level. The download by project feature allows retrieval of data from individual BioProjects. In addition, the latest annotation and latest transcriptome assembly options provide direct access to the study's GFF3 annotation and FASTA transcriptome assembly files, respectively, supporting comprehensive downstream analyses.

A compelling case study focusing on 3-hydroxy-3-methylglutaryl-CoA reductase, a key enzyme in the artemisinin biosynthesis pathway (Li et al., 2024), illustrates the utility of ArtemisiaDB (Supplemental Figures 2 and 3). Co-expression analysis in leaf tissue identified 11 hormone-responsive genes, eight of which were TFs belonging to the auxin response factor (ARF) family. This finding suggests a novel regulatory link between auxin signaling and artemisinin biosynthesis. Although this finding is correlative, increasing experimental evidence supports a role for auxin signaling—and ARFs in particular—in both trichome development and artemisinin biosynthesis in *A. annua*. For example, overexpression of the auxin biosynthesis gene *iaaM* significantly increases the density, length, and width of glandular secretory trichomes, along with elevated artemisinin content, suggesting that auxin promotes both trichome development and the metabolic pathways involved in artemisinin production (Zhao et al., 2024). In addition, the miR160–ARF1 regulatory module has been shown to control glandular secretory trichome formation and activate the expression of *DBR2*, a key gene in the artemisinin biosynthesis pathway, suggesting that ARFs function as upstream regulators of both trichome development and metabolite production (Guo et al., 2023). A plausible mechanism is that ARFs bind to auxin-responsive promoter elements upstream of biosynthetic genes such as *HMGR* or *DBR2*, thereby linking hormonal signaling to transcriptional regulation. Future validation using chromatin immunoprecipitation, promoter–reporter assays, or functional analyses of ARF overexpression and silencing lines will be essential to confirm these interactions. This case study demonstrates how ArtemisiaDB can be used to generate testable hypotheses and identify regulatory components to guide metabolic engineering strategies for enhanced artemisinin production.

Looking ahead, ArtemisiaDB is designed to support future multi-omics expansion. Planned updates include the integration of single-cell and spatial transcriptomics, which will enable cell-type-specific exploration of biosynthetic pathways, as well as epigenomic layers—such as DNA methylation and histone modification data—to better elucidate the regulatory mechanisms underlying gene expression. Moreover, the incorporation of metabolite flux and isotope-labeling data will allow quantitative integration of transcriptomic and metabolomic dynamics. These developments will extend ArtemisiaDB from a descriptive resource toward a more predictive framework for understanding and engineering *A. annua* metabolism.

(C) BUSCO summary assessing assembly completeness. ST, StringTie; Ref. Curated, manually curated reference transcriptome; Ref. Trans., reference transcriptome; ST Clustered, StringTie assemblies clustered with MMseqs2; ST SMRT, StringTie assemblies integrating SMRT long-read data.

(D) t-distributed stochastic neighbor embedding (t-SNE) plot of RNA-seq samples based on the top 14 principal components for dimensionality reduction.

(E) Distribution of genes across four expression categories defined by the τ index and median TPM values: null expression (median TPM < 1 in all tissues), weak expression (median TPM < 5 in all tissues), broad expression (τ < 0.85 and median TPM > 5), and tissue-specific expression ($\tau \geq$ 0.85 and median TPM > 5).

(F) UpSet plot showing the overlap of tissue-specific genes across tissues.

(G) Heatmap showing the distribution of transcription factor (TF) families among tissue-specific genes identified using the τ index. Numbers indicate the counts of TFs in each family exhibiting tissue-specific expression across five tissues (petiole, leaf, stem, flower, and root). TF families were classified based on PlantTFDB and Pfam annotations.

In conclusion, ArtemisiaDB provides a valuable resource for advancing our understanding of the molecular mechanisms governing artemisinin biosynthesis in *A. annua*. By providing an integrated platform for high-quality multi-omics data, ArtemisiaDB facilitates efforts to optimize artemisinin production and advance plant metabolic engineering. We believe this database will be instrumental for researchers working on both fundamental and applied research on *A. annua*.

DATA AND CODE AVAILABILITY

The datasets underpinning this study are available in the aforementioned online repositories. All code used for the analyses is accessible via the following GitHub repository: https://github.com/ayattaheri/artemisia-db. ArtemisiaDB (https://artemisia-db.com) will be updated annually to incorporate newly published RNA-seq, metabolomic, and functional datasets, with each update version documented on the database website.

FUNDING

This work was supported by the Shanghai Municipal Science and Technology Major Project; the National Natural Science Foundation of China (82274047); the Bill & Melinda Gates Foundation (INV-027291); and the National Key Research and Development Program of China (2023YFC3503901).

ACKNOWLEDGMENTS

In accordance with the grant conditions of the Bill & Melinda Gates Foundation, any author-accepted manuscript arising from this submission will be made available under a Creative Commons Attribution 4.0 Generic License. The computations presented in this study were performed on the Siyuan-1 cluster, supported by the Center for High Performance Computing at Shanghai Jiao Tong University. We acknowledge the valuable contributions of the researchers and organizations who generated the genome and RNA-seq data that made this study possible. The authors declare no competing interests.

AUTHOR CONTRIBUTIONS

A.T. conceived the study, performed data analyses, developed the web application, and drafted the manuscript. F.A.-S. contributed to data analysis, manuscript revision, and discussion. X.F. and K.T. provided funding support. K.T. supervised the study and serves as the corresponding author. Y.Z., L.L., and Y.W. contributed to manuscript revision and discussion. All authors reviewed and approved the final manuscript.

SUPPLEMENTAL INFORMATION

Supplemental information is available at *Plant Communications Online*.

Received: July 25, 2025
Revised: January 13, 2026
Accepted: March 16, 2026
Published: March 17, 2026

Ayat Taheri[1,2], Fabricio Almeida-Silva[3,4], Yaojie Zhang[1], Xueqing Fu[5], Ling Li[1], Yuliang Wang[1,6] and Kexuan Tang[1,7,]*

[1]Joint International Research Laboratory of Metabolic and Developmental Sciences, Frontiers Science Center for Transformative Molecules, Plant Biotechnology Research Center, Fudan-SJTU-Nottingham Plant Biotechnology R&D Center, School of Agriculture and Biology, Shanghai Jiao Tong University, Shanghai 200240, China
[2]College of Life Sciences and Medicine, Key Laboratory of Plant Secondary Metabolism and Regulation of Zhejiang Province, Zhejiang Sci-Tech University, Hangzhou 310018, China
[3]Department of Plant Biotechnology and Bioinformatics, Ghent University, 9052 Ghent, Belgium
[4]VIB Center for Plant Systems Biology, VIB, 9052 Ghent, Belgium
[5]School of Design, Shanghai Jiao Tong University, Shanghai 200240, China
[6]Shanghai Jiao Tong University Sichuan Research Institute, Chengdu 610213, China
[7]Yazhouwan National Laboratory, No. 8 Huanjin Road, Yazhou District, Sanya City 572024, China
***Correspondence: Kexuan Tang (kxtang@sjtu.edu.cn)**
https://doi.org/10.1016/j.xplc.2026.101826

REFERENCES

Almeida-Silva, F., Pedrosa-Silva, F., and Venancio, T.M. (2023). The Soybean Expression Atlas v2: A comprehensive database of over 5000 RNA-seq samples. Plant J. **116**:1041–1051. https://doi.org/10.1111/tpj.16459.

Chen, T., Yang, M., Cui, G., Tang, J., Shen, Y., Liu, J., Yuan, Y., Guo, J., and Huang, L. (2024). IMP: bridging the gap for medicinal plant genomics. Nucleic Acids Res. **52**:D1347–D1354. https://doi.org/10.1093/nar/gkad898.

Guo, Z., Hao, K., Lv, Z., Yu, L., Bu, Q., Ren, J., Zhang, H., Chen, R., and Zhang, L. (2023). Profiling of phytohormone-specific microRNAs and characterization of the miR160-ARF1 module involved in glandular trichome development and artemisinin biosynthesis in *Artemisia annua*. Plant Biotechnol. J. **21**:591–605. https://doi.org/10.1111/pbi.13974.

Hassani, D., Taheri, A., Fu, X., Qin, W., Hang, L., Ma, Y., and Tang, K. (2023). Elevation of artemisinin content by co-transformation of artemisinin biosynthetic pathway genes and trichome-specific transcription factors in *Artemisia annua*. Front. Plant Sci. **14**:1118082. https://doi.org/10.3389/fpls.2023.1118082.

Li, Y., Yang, Y., Li, L., Tang, K., Hao, X., and Kai, G. (2024). Advanced metabolic engineering strategies for increasing artemisinin yield in *Artemisia annua* L. Hortic. Res. **11**:uhad292. https://doi.org/10.1093/hr/uhad292.

Liao, B., Shen, X., Xiang, L., Guo, S., Chen, S., Meng, Y., Liang, Y., Ding, D., Bai, J., Zhang, D., et al. (2022). Allele-aware chromosome-level genome assembly of *Artemisia annua* reveals the correlation between ADS expansion and artemisinin yield. Mol. Plant **15**:1310–1328. https://doi.org/10.1016/j.molp.2022.05.013.

Liu, J., Zhang, Y., Zheng, Y., Zhu, Y., Shi, Y., Guan, Z., Lang, K., Shen, D., Huang, W., and Dou, D. (2023). PlantExp: a platform for exploration of gene expression and alternative splicing based on public plant RNA-seq samples. Nucleic Acids Res. **51**:D1483–D1491. https://doi.org/10.1093/nar/gkac917.

Mirdita, M., Steinegger, M., and Söding, J. (2019). MMseqs2 desktop and local web server app for fast, interactive sequence searches. Bioinformatics **35**:2856–2858. https://doi.org/10.1093/bioinformatics/bty1057.

Pertea, M., Pertea, G.M., Antonescu, C.M., Chang, T.-C., Mendell, J.T., and Salzberg, S.L. (2015). StringTie enables improved reconstruction of a transcriptome from RNA-seq reads. Nat. Biotechnol. **33**:290–295. https://doi.org/10.1038/nbt.3122.

Prjibelski, A.D., Mikheenko, A., Joglekar, A., Smetanin, A., Jarroux, J., Lapidus, A.L., and Tilgner, H.U. (2023). Accurate isoform discovery with IsoQuant using long reads. Nat. Biotechnol. **41**:915–918. https://doi.org/10.1038/s41587-022-01565-y.

Venturini, L., Caim, S., Kaithakottil, G.G., Mapleson, D.L., and Swarbreck, D. (2018). Leveraging multiple transcriptome assembly methods for improved gene structure annotation. GigaScience **7**:giy093. https://doi.org/10.1093/gigascience/giy093.

Zhao, Y., Xiao, N., Long, Y., Luo, J., Liu, X., and Zhang, X. (2024). Effects of co-overexpression of *ALDH1, CYP71AV1* , and *iaaM* on the density, length, and width of glandular secretory trichomes and the contents of artemisinin in *Artemisia annua*. Can. J. Plant Sci. **104**:161–167. https://doi.org/10.1139/cjps-2023-0119.

Plant Communications, Volume 7

Supplemental information

ArtemisiaDB: A comprehensive multi-omics database for *Artemisia annua*

Ayat Taheri, Fabricio Almeida-Silva, Yaojie Zhang, Xueqing Fu, Ling Li, Yuliang Wang, and Kexuan Tang

ArtemisiaDB: A Comprehensive Multi-Omics Database for *Artemisia annua*

Ayat Taheri[1,2], Fabricio Almeida-Silva[3,4], Yaojie Zhang[1], Xueqing Fu[5], Ling Li[1], Yuliang Wang[1,6], Kexuan Tang[1,7*]

1. Joint International Research Laboratory of Metabolic and Developmental Sciences, Frontiers Science Center for Transformative Molecules, Plant Biotechnology Research Center, Fudan-SJTU-Nottingham Plant Biotechnology R&D Center, School of Agriculture and Biology, Shanghai Jiao Tong University, Shanghai 200240, China
2. College of Life Sciences and Medicine, Key Laboratory of Plant Secondary Metabolism and Regulation of Zhejiang Province, Zhejiang Sci-Tech University, Hangzhou 310018, China
3. Department of Plant Biotechnology and Bioinformatics, Ghent University, 9052 Ghent, Belgium
4. VIB Center for Plant Systems Biology, VIB, 9052 Ghent, Belgium
5. School of Design, Shanghai Jiao Tong University, Shanghai 200240, China
6. Shanghai Jiao Tong University Sichuan Research Institute, Chengdu, 610213, China
7. Yazhouwan National Laboratory, No. 8 Huanjin Road, Yazhou District, Sanya City 572024, China

***Corresponding author:**

Kexuan Tang, E-mail: kxtang@sjtu.edu.cn

Methods

Data Sources and Preprocessing

For building the ArtemisiaDB, SRA files of RNA-Seq data available on the NCBI's Sequence Read Archive (SRA), National Genomics Data Center (https://ngdc.cncb.ac.cn/) and Global Pharmacopeia Genome Database (http://www.gpgenome.com/) databases up to 2025-10-01 (317 samples) along with the latest available genome of *A. annua* (LQ9-Phase0) (Liao et al., 2022) were downloaded (Supplemental table 1). Gene expression profiling was conducted using a combination of paired-end and single-end short-read RNA-seq data. To improve the accuracy of our transcriptome assembly, we integrated PacBio Iso-Seq full-length transcript reads. Although long-read sequencing is typically employed for genome assembly, Iso-Seq data were specifically used here to capture complete transcript models and improve isoform resolution.

Conversion of SRA files to FASTQ was performed using fastq-dump (3.1.0), which is part of the SRA Toolkit. To ensure the quality of the FASTQ files, fastp (0.23.2) (Chen et al., 2018) was used, and FASTQ files with a mean read length lower than 40 and/or Q20 rate lower than 80%, and the adapter sequences were removed.

Transcriptome Assembly

To construct a comprehensive transcriptome for *A. annua*, we integrated both short-read and long-read RNA-Seq data. Short-read sequences were aligned using HISAT2 (v2.2.1), while long-read sequences were aligned with Minimap2 (v2.17). Samples with an overall alignment rate below 50% were excluded from further analysis.

For transcript assembly, StringTie (v2.2.1) (Pertea et al., 2015) was used for short-read data, while both StringTie and IsoQuant were employed for long-read data. The assembled transcripts from short-read sequences were merged using the `stringtie --merge` command to generate a unified transcript set.

Initial quality assessment of the assembled transcriptomes

The quality of the resulting transcriptomes was evaluated to identify the most comprehensive and accurate assembly for downstream analysis. Four assemblies were compared: the reference transcriptome, the StringTie-assembled transcriptome derived from both short-read and long-read RNA-Seq data, and the IsoQuant-assembled transcriptome. To assess assembly completeness and quality, BUSCO (embryophyta_odb10 database, v5.7.1) (Seppey et al., 2019) was used, which evaluates transcriptome assemblies based on the presence of conserved single-

copy orthologs. This initial evaluation provided a foundational understanding of the quality of each assembly, which was essential for developing a scoring system in the Mikado pipeline. By identifying a robust and representative transcriptome, this approach ensured not only a comprehensive assembly but also a framework for prioritizing transcripts and minimizing redundancy in subsequent analyses.

Clustering Using MMseqs2

While the StringTie-assembled transcriptome exhibited the highest number of complete BUSCOs and the lowest number of missing BUSCOs, it also displayed a high number of duplications. This indicated a significant level of redundancy within the assembly. To address this issue, MMseqs2 (Many-against-Many sequence searching tool) was employed to cluster highly similar sequences and reduce redundancy (Steinegger and Söding, 2017). By grouping similar transcripts (--min-seq-id 0.8), the clustering process aimed to refine the assembly, ensuring a more accurate representation of unique genes while maintaining the integrity of biologically relevant sequences.

Splice-site filtering

To improve the accuracy of splice-site detection in the long-read assemblies, Portcullis was employed. Portcullis is a specialized tool designed to identify and filter splice junctions with high precision, addressing errors commonly found in transcriptome assemblies generated from long-read sequencing data (Mapleson et al., 2018). By applying this filtering step, erroneous or low-confidence splice sites were removed, resulting in a cleaner and more reliable set of splice junctions. This refinement was crucial for ensuring the quality of the transcriptome assembly and for minimizing false positives that could impact downstream analyses, including transcript annotation and functional characterization.

Merging annotations using the Mikado pipeline

To integrate and refine the four transcriptome assemblies—reference transcriptome, StringTie (short-read and long-read), and IsoQuant—we used the Mikado pipeline (Venturini et al., 2018). Mikado is designed to identify the most accurate and representative set of transcripts from multiple assembly methods. The pipeline first defines gene loci based on overlap criteria and evaluates each transcript within a locus using up to 50 metrics, including ORF and cDNA size, UTR length, and the relative position of the ORF. Mikado also utilizes BLASTX-based protein similarity and can integrate junction confidence data from Portcullis to improve splice-site accuracy. The best-scoring transcripts are selected as primary gene models, while valid splice

variants are retained when compatible with the primary isoform. This approach ensures that the final set of transcripts minimizes redundancy, improves gene model quality, and enhances the representation of expressed loci. Mikado's ability to integrate data from both short-read and long-read technologies allowed us to merge assemblies generated through different approaches, resulting in a more comprehensive and accurate transcriptome for further downstream analysis.

Mikado Assembly Validation

To evaluate the Mikado-based assembly, we benchmarked its performance against the reference and manually-curated transcriptomes. Biological completeness was assessed using BUSCO v5.7.1 against the embryophyta_odb10 lineage, while structural continuity and ORF content were analyzed using Transrate (Smith-Unna et al., 2016). Functional depth was quantified by identifying transcripts with ≥ 90% protein coverage via Trinity (Grabherr et al., 2011), and assembly accuracy was validated through rnaQUAST (Bushmanova et al., 2016)metrics and Reads Mapped Back (RMBT) percentages (Langmead and Salzberg, 2012). Overall, the Mikado assembly demonstrated superior quality across nearly all evaluated metrics, justifying its selection as the core reference for ArtemisiaDB (Supplemental Table 4).

Gene-level transcript abundance

After generating the representative assembly for *A. annua*, transcript abundances were estimated using the 'mapping-based' mode of Salmon (v1.10.0) with the '--dumpEq' option, which produces the equivalence classes that can be used in differential transcript usage analysis (Patro et al., 2017). Variations in library size across samples are taken into consideration by transcripts per million (TPM). However, TPM does not take variations in transcript length into consideration, meaning that, even when transcripts are expressed at the same level, longer transcripts will typically have higher TPM values than shorter transcripts. To obtain the gene-level transcript abundances, the bias-corrected counts without an offset method from tximport R package was used (Soneson et al., 2016). The "lengthScaledTPM" argument in tximport adjusts for differences in both library size and transcript length across samples. For the transcript-level transcript abundance estimates, the "dtuScaledTPM" argument was used, which first scales using the median transcript length among isoforms of a gene, and then by library size.

Dimensionality reduction

Because bioinformatics data contains numerous attributes (variables), they are naturally high-dimensional. This high dimensionality can present major hurdles for data analysis, obscuring valuable biological insights and complicating downstream analysis. Therefore, appropriate procedures should be taken to boost the biological significance of the data by reducing the number of attributes using dimensionality reduction techniques (Cao et al., 2020). For transcriptomics in particular, dimensionality reduction helps to mitigate noise and enhance the signal from the biological data. To achieve this goal, techniques such as principal component analysis (PCA), t-distributed stochastic neighbor embedding (t-SNE) (Van der Maaten and Hinton, 2008), and uniform manifold approximation and projection (UMAP) (McInnes et al., 2020) are commonly employed. In the present study, we used scran R package (Lun et al., 2016). After log-transforming the gene counts normalized by library size, we extracted the top 3000 genes with the highest biological components to model the mean-variance relationships. Following performing PCA analysis, 14 principal components, which accounted for 70% of the variation, were selected for dimensionality reduction using the t-SNE and UMAP algorithms. Six different perplexity values (5, 10, 15, 20, 25, 30) for t-SNE and six different numbers of nearest neighbors (10, 20, 30, 40, 50, 60) for UMAP were tested. The optimal values were determined based on visual inspection (Supplemental Figure 4A-C).

Identification of broadly expressed and tissue-specific genes

Identifying broadly expressed and tissue-specific genes is crucial for understanding gene function and regulation. Broadly expressed genes often play essential roles in basic cellular processes, while tissue-specific genes are typically involved in specialized tissue functions. Analyzing these expression patterns helps researchers understand gene functions and regulatory mechanisms (Das and Bansal, 2019). To achieve this, we calculated the tissue specificity index (τ index) for each gene by using a log-transformed matrix of the median TPM values per tissue (Yanai et al., 2005). Based on the criteria established by Lüleci and Yılmaz (2022), genes were categorized into four groups according to their τ index and median TPM values: (1) Null expression: genes with median TPM < 1 in all tissues, indicating no expression; (2) Weak expression: genes with median TPM < 5 in all tissues; (3) Broad expression: genes with $\tau < 0.85$ and median TPM > 5; and (4) Tissue-specific expression: genes with $\tau \geq 0.85$ and median TPM > 5 (Supplemental Figure 4D).

Functional annotation of the *A. annua* transcriptome

To create a unified database for the functional annotation of the *A. annua* transcriptome, several analyses were performed. Transcription factors (TFs) were identified using two approaches: first, by performing a BLASTx search against a collection of TFs from the PlantTFDB (Jin et al., 2017), and second, by extracting PFAM IDs related to 63 different TF families through InterProScan (Supplemental Figure 5). For the BLASTx analysis, Diamond was used with the following parameters: --ultra-sensitive --id 60 --evalue 1e-5 --min-score 150 (Buchfink et al., 2021). EggNOG-mapper (2.1.12) (Cantalapiedra et al., 2021) using the HMMER3 mode was used to annotate orthologous groups and functional categories. InterPro domains, Gene Ontology (GO) terms, and KEGG pathways were assigned using InterProScan (5.67-99.0) (Supplemental Figure 6); The KEGG ORTHOLOGY (KO) database (https://www.genome.jp/kegg/ko.html) was used to obtain KO descriptions. Additionally, BLASTx against *Arabidopsis thaliana* protein-coding genes (Araport11 release, 2022-09-15) and the Uniprot database (release 01.03.2023) was performed to further enhance the functional characterization of the transcriptome. To facilitate genome editing, we integrated a design tool powered by the CrisprDesign R package (1.12.0), which generates high-fidelity CRISPR sgRNA candidates with optimized targeting parameters (Hoberecht et al., 2022). A list of artemisinin-related genes was obtained from recently published research (Li et al., 2024). Their sequences were retrieved from the NCBI database, and the corresponding gene IDs in the current annotation were identified using BLASTn for further analysis. For enrichment analysis, enricher() function of the clusterProfiler package was utilized, with all expressed genes serving as the background set.

Development of the web application

The web application was developed using the Shiny framework (Chang et al., 2021), with a Bootstrap-based layout. A MySQL database was used to manage data associated with various sections of the application, ensuring well-organized data retrieval and storage. A partitioned Parquet directory was employed to store the expression database, with the arrow R package (Richardson et al., 2022) providing an efficient interface between R and the Apache Arrow platform. For data visualization, the application leveraged the ggplot2 (Wickham, 2016), plotly (Inc, 2015), and ComplexHeatmap (Gu, 2022) packages.

References

Buchfink, B., Reuter, K., and Drost, H.-G. (2021). Sensitive protein alignments at tree-of-life scale using DIAMOND. *Nat. Methods* **18**:366–368. https://doi.org/10.1038/s41592-021-01101-x.

Bushmanova, E., Antipov, D., Lapidus, A., Suvorov, V., and Prjibelski, A. D. (2016). rnaQUAST: a quality assessment tool for de novo transcriptome assemblies. *Bioinforma. Oxf. Engl.* **32**:2210–2212. https://doi.org/10.1093/bioinformatics/btw218.

Cantalapiedra, C. P., Hernández-Plaza, A., Letunic, I., Bork, P., and Huerta-Cepas, J. (2021). eggNOG-mapper v2: Functional Annotation, Orthology Assignments, and Domain Prediction at the Metagenomic Scale. *Mol. Biol. Evol.* **38**:5825–5829. https://doi.org/10.1093/molbev/msab293.

Cao, Y., Geddes, T. A., Yang, J. Y. H., and Yang, P. (2020). Ensemble deep learning in bioinformatics. *Nat. Mach. Intell.* **2**:500–508. https://doi.org/10.1038/s42256-020-0217-y.

Chang, W., Cheng, J., Allaire, J., Sievert, C., Schloerke, B., Xie, Y., Allen, J., McPherson, J., Dipert, A., and Borges, B. (2021). shiny: Web Application Framework for R. 2021. R package version 1.6. 0. *Ref. Source* Advance Access published 2021.

Chen, S., Zhou, Y., Chen, Y., and Gu, J. (2018). fastp: an ultra-fast all-in-one FASTQ preprocessor. *Bioinformatics* **34**:i884–i890. https://doi.org/10.1093/bioinformatics/bty560.

Das, S., and Bansal, M. (2019). Variation of gene expression in plants is influenced by gene architecture and structural properties of promoters. *PLoS ONE* **14**:e0212678. https://doi.org/10.1371/journal.pone.0212678.

Grabherr, M. G., Haas, B. J., Yassour, M., Levin, J. Z., Thompson, D. A., Amit, I., Adiconis, X., Fan, L., Raychowdhury, R., Zeng, Q., et al. (2011). Trinity: reconstructing a full-length transcriptome without a genome from RNA-Seq data. *Nat. Biotechnol.* **29**:644–652. https://doi.org/10.1038/nbt.1883.

Gu, Z. (2022). Complex heatmap visualization. *iMeta* **1**:e43. https://doi.org/10.1002/imt2.43.

Hoberecht, L., Perampalam, P., Lun, A., and Fortin, J.-P. (2022). A comprehensive Bioconductor ecosystem for the design of CRISPR guide RNAs across nucleases and technologies. *Nat. Commun.* **13**:6568. https://doi.org/10.1038/s41467-022-34320-7.

Inc, P. (2015). Collaborative data science, Montreal, QC: Plotly Technologies Inc Advance Access published 2015.

Jin, J., Tian, F., Yang, D.-C., Meng, Y.-Q., Kong, L., Luo, J., and Gao, G. (2017). PlantTFDB 4.0: toward a central hub for transcription factors and regulatory interactions in plants. *Nucleic Acids Res.* **45**:D1040–D1045. https://doi.org/10.1093/nar/gkw982.

Langmead, B., and Salzberg, S. L. (2012). Fast gapped-read alignment with Bowtie 2. *Nat. Methods* **9**:357–359. https://doi.org/10.1038/nmeth.1923.

Li, Y., Yang, Y., Li, L., Tang, K., Hao, X., and Kai, G. (2024). Advanced metabolic engineering strategies for increasing artemisinin yield in *Artemisia annua* L. *Hortic. Res.* **11**:uhad292. https://doi.org/10.1093/hr/uhad292.

Liao, B., Shen, X., Xiang, L., Guo, S., Chen, S., Meng, Y., Liang, Y., Ding, D., Bai, J., Zhang, D., et al. (2022). Allele-aware chromosome-level genome assembly of *Artemisia annua* reveals the correlation between ADS expansion and artemisinin yield. *Mol. Plant* **15**:1310–1328. https://doi.org/10.1016/j.molp.2022.05.013.

Lüleci, H. B., and Yılmaz, A. (2022). Robust and rigorous identification of tissue-specific genes by statistically extending tau score. *BioData Min.* **15**:31. https://doi.org/10.1186/s13040-022-00315-9.

Lun, A. T. L., McCarthy, D. J., and Marioni, J. C. (2016). A step-by-step workflow for low-level analysis of single-cell RNA-seq data with Bioconductor. *F1000Research* **5**:2122. https://doi.org/10.12688/f1000research.9501.2.

Mapleson, D., Venturini, L., Kaithakottil, G., and Swarbreck, D. (2018). Efficient and accurate detection of splice junctions from RNA-seq with Portcullis. *GigaScience* **7**:giy131. https://doi.org/10.1093/gigascience/giy131.

McInnes, L., Healy, J., and Melville, J. (2020). UMAP: Uniform Manifold Approximation and Projection for Dimension Reduction Advance Access published September 17, 2020.

Patro, R., Duggal, G., Love, M. I., Irizarry, R. A., and Kingsford, C. (2017). Salmon provides fast and bias-aware quantification of transcript expression. *Nat. Methods* **14**:417–419. https://doi.org/10.1038/nmeth.4197.

Pertea, M., Pertea, G. M., Antonescu, C. M., Chang, T.-C., Mendell, J. T., and Salzberg, S. L. (2015). StringTie enables improved reconstruction of a transcriptome from RNA-seq reads. *Nat. Biotechnol.* **33**:290–295. https://doi.org/10.1038/nbt.3122.

Richardson, N., Cook, I., Crane, N., Keane, J., François, R., and Ooms, J. (2022). Arrow: integration to apache arrow Advance Access published 2022.

Seppey, M., Manni, M., and Zdobnov, E. M. (2019). BUSCO: assessing genome assembly and annotation completeness. In *Gene prediction*, pp. 227–245. Springer.

Smith-Unna, R., Boursnell, C., Patro, R., Hibberd, J. M., and Kelly, S. (2016). TransRate: reference-free quality assessment of de novo transcriptome assemblies. *Genome Res.* **26**:1134–1144. https://doi.org/10.1101/gr.196469.115.

Soneson, C., Love, M. I., and Robinson, M. D. (2016). Differential analyses for RNA-seq: transcript-level estimates improve gene-level inferences. *F1000Research* **4**:1521. https://doi.org/10.12688/f1000research.7563.2.

Steinegger, M., and Söding, J. (2017). MMseqs2 enables sensitive protein sequence searching for the analysis of massive data sets. *Nat. Biotechnol.* **35**:1026–1028. https://doi.org/10.1038/nbt.3988.

Van der Maaten, L., and Hinton, G. (2008). Visualizing data using t-SNE. *J. Mach. Learn. Res.* **9**.

Venturini, L., Caim, S., Kaithakottil, G. G., Mapleson, D. L., and Swarbreck, D. (2018). Leveraging multiple transcriptome assembly methods for improved gene structure annotation. *GigaScience* **7**. https://doi.org/10.1093/gigascience/giy093.

Wickham, H. (2016). Getting Started with ggplot2. In *ggplot2*, pp. 11–31. Cham: Springer International Publishing.

Supplemental Figures

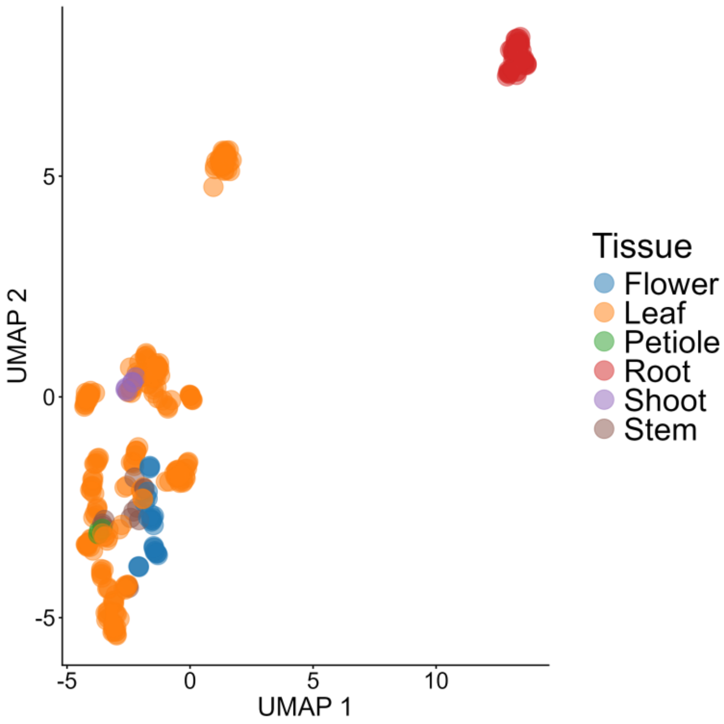

Supplemental Figure 1. UMAP plot of RNA-seq samples based on the top 14 principal components for dimensionality reduction.

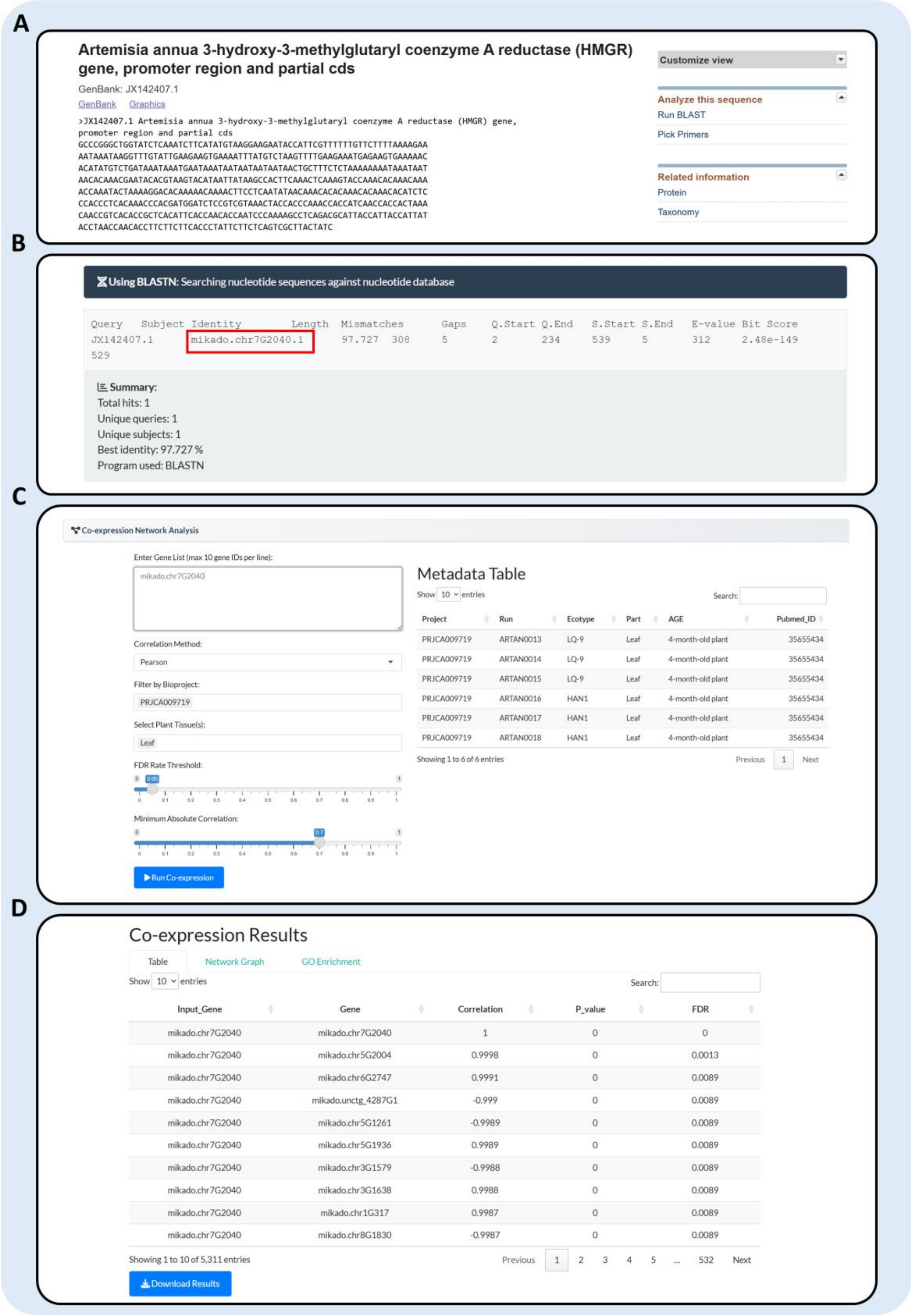

Supplemental Figure 2. Case study of the *HMGR* gene in ArtemisiaDB (Part 1). (A) HMGR gene sequence from NCBI. (B) BLAST results against ArtemisiaDB to identify the corresponding gene ID. (C) Selection of co-expression analysis parameters (Pearson method, leaf samples, FDR ≤ 0.05, |correlation| ≥ 0.7). (D) Co-expression results table.

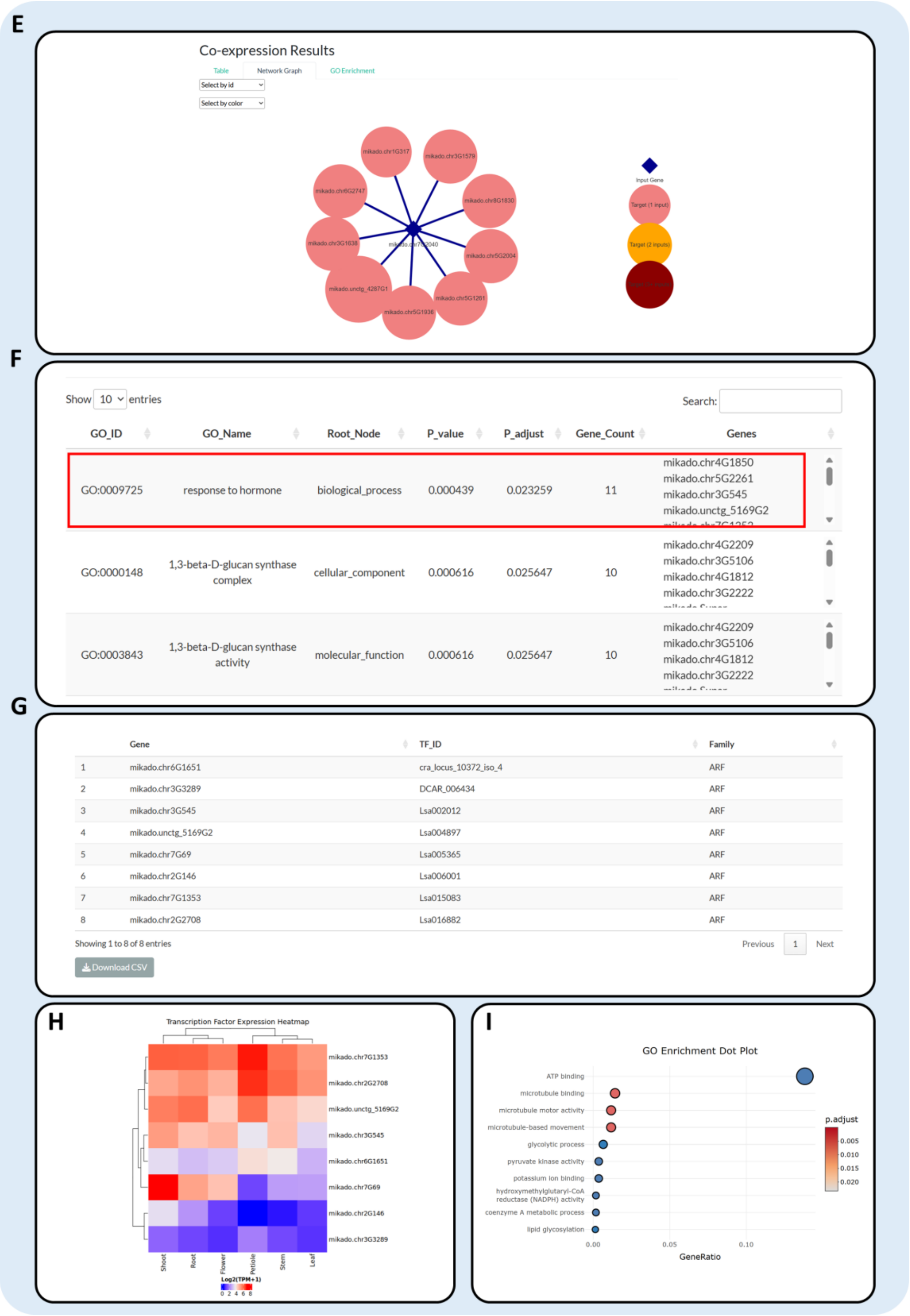

Supplemental Figure 3. Case study of the *HMGR* gene in ArtemisiaDB (Part 2). (E) Co-expression network of the input gene (*HMGR*) and its top 10 co-expressed genes. (F) GO enrichment analysis of the co-expressed genes, highlighting the "response to hormone" term. (G) TFDB search of the co-expressed genes, revealing several ARF transcription factors. (H) Heatmap showing the expression of the identified ARF transcription factors across different tissues. (I) GO enrichment dot plot of the co-expressed genes, showing the most significantly enriched biological process terms.

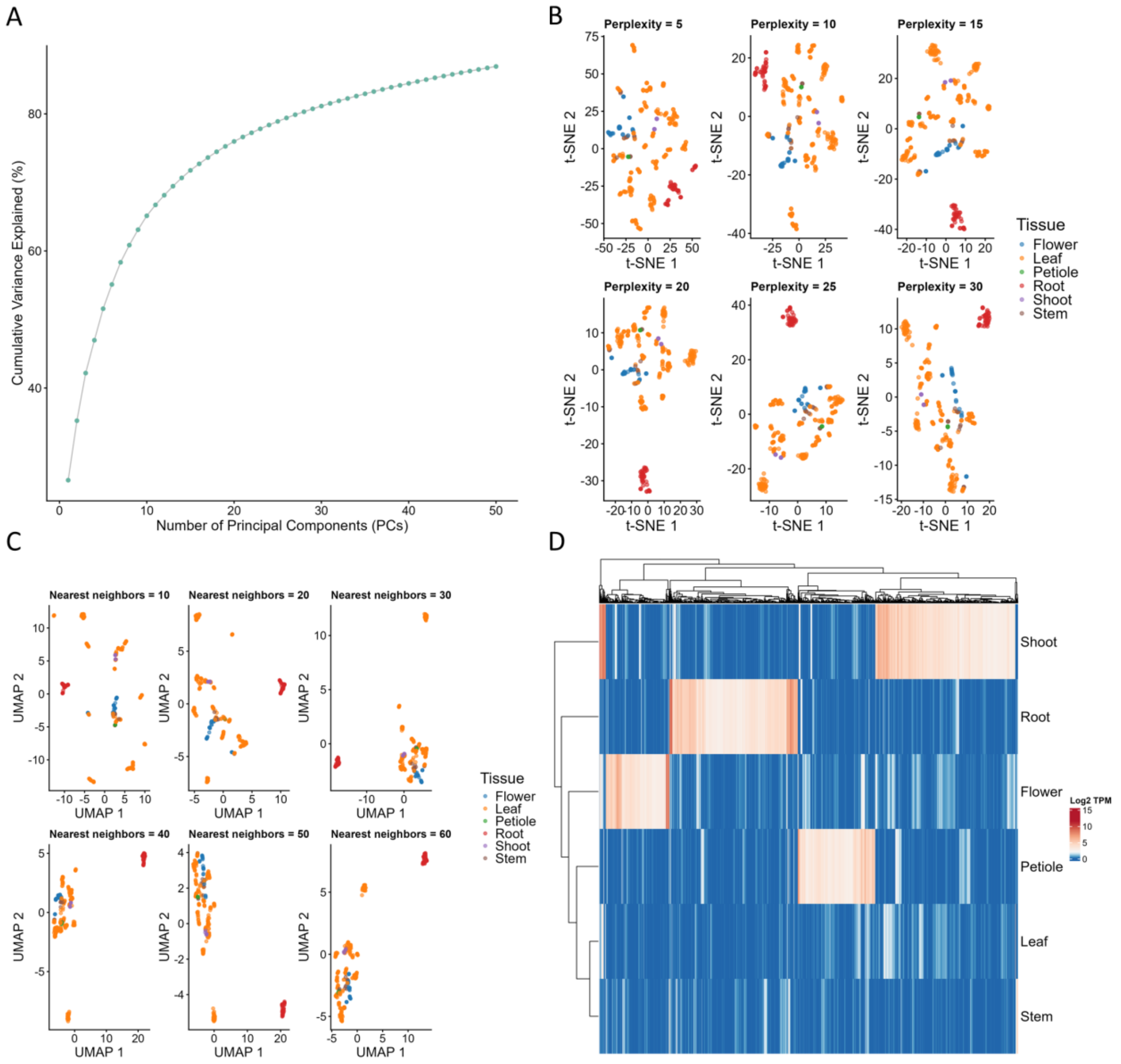

Supplemental Figure 4. (A) Cumulative variance explained by each principal component (PC) in the transcriptome analysis. (B) t-SNE plots generated using different perplexity values (5, 10, 15, 20, 25, 30). (C) UMAP plots based on varying numbers of nearest neighbors (10, 20, 30, 40, 50, 60). (D) Heatmap of median expression levels of tissue-specific genes across different tissues.

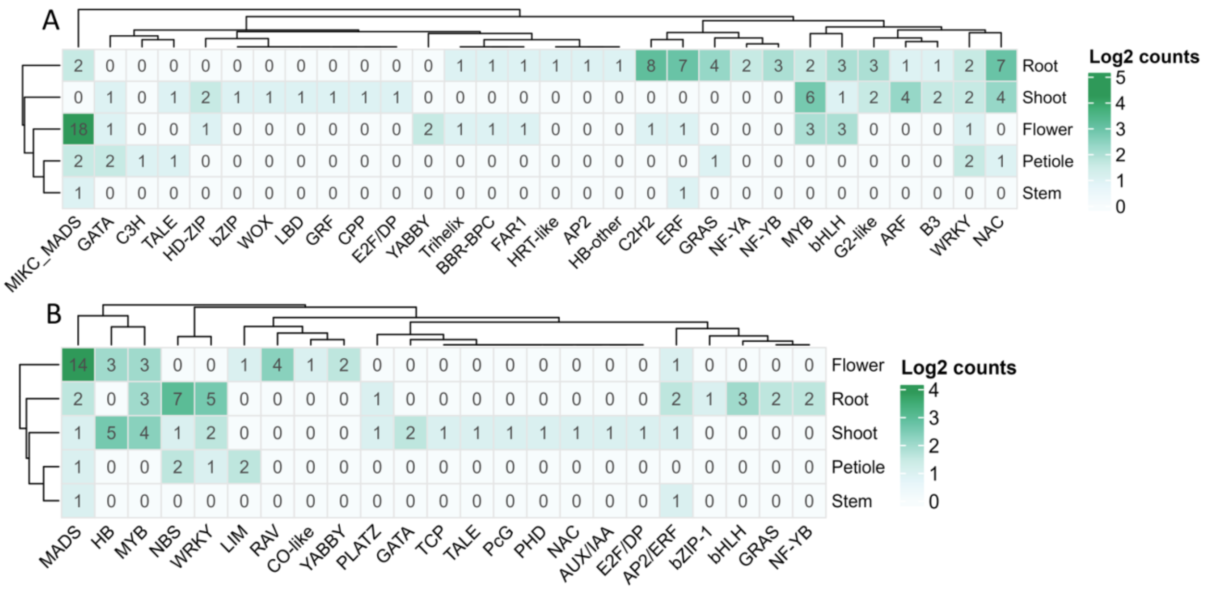

Supplemental Figure 5. Heatmap of transcription factor distribution across tissue-specific genes identified from PlantTFDB. (B) Heatmap of transcription factor distribution across tissue-specific genes identified using Pfam IDs.

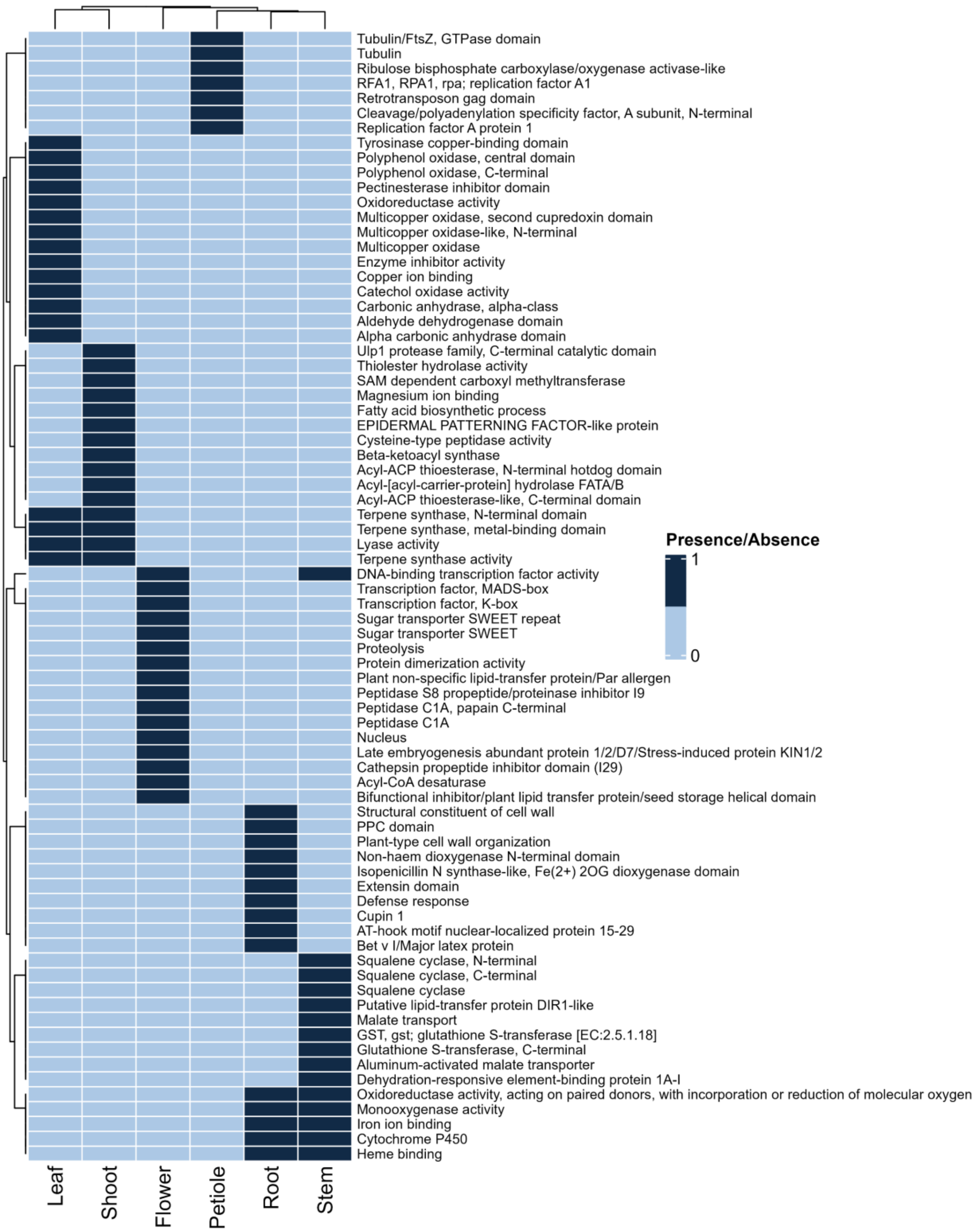

Supplemental Figure 6. Heatmap of Gene Ontology (GO) terms and InterPro domain enrichment for tissue-specific genes.
